# The effect of social relationships on cognitive decline in older adults: an updated systematic review and meta-analysis of longitudinal cohort studies

**DOI:** 10.1186/s12889-022-12567-5

**Published:** 2022-02-11

**Authors:** Matteo Piolatto, Federico Bianchi, Matteo Rota, Alessandra Marengoni, Aliakbar Akbaritabar, Flaminio Squazzoni

**Affiliations:** 1 Cluster of Excellence, Department of Sociology, University of Konstanz, Universität-Str. 10, Konstanz, Germany; 2 Department of Social and Political Sciences, University of Milan, Via Conservatorio 7 20122, Milan, Italy; 3 Department of Molecular and Translational Medicine, University of Brescia, Viale Europa 11, Brescia, Italy; 4 Department of Clinical and Experimental Sciences, University of Brescia, Viale Europa 11, Brescia, Italy; 5 Laboratory of Digital and Computational Demography, Max Planck Institute for Demographic Research, Konrad-Zuse-Str. 1, Rostock, Germany

**Keywords:** Social relationships, Cognitive decline, Social networks, Systematic review, Meta-analysis

## Abstract

**Background:**

A previous meta-analysis (Kuiper et al., 2016) has shown that multiple aspects of social relationships are associated with cognitive decline in older adults. Yet, results indicated possible bias in estimations of statistical effects due to the heterogeneity of study design and measurements. We have updated this meta-analysis adding all relevant publications from 2012 to 2020 and performed a cumulative meta-analysis to map the evolution of this growing field of research (+80% of studies from 2012-2020 compared to the period considered in the previous meta-analysis).

**Methods:**

Scopus and Web of Science were searched for longitudinal cohort studies examining structural, functional and combined effects of social relationships. We combined Odds Ratios (OR) with 95% confidence intervals (CI) using random effects meta-analysis and assessed sources of heterogeneity and the likelihood of publication bias. The risk of bias was evaluated with the Quality of Prognosis Studies in Systematic Reviews (QUIPS) tool.

**Results:**

The review was prospectively registered on PROSPERO (ID: CRD42019130667). We identified 34 new articles published in 2012-2020. Poor social relationships were associated with cognitive decline with increasing precision of estimates compared to previously reviewed studies [(for structural, 17 articles, OR: 1.11; 95% CI: 1.08; 1.14) (for functional, 16 articles, OR: 1.12; 95% CI: 1.05; 1.20) (for combined, 5 articles, OR: 1.15; 95% CI: 1.06; 1.24)]. Meta-regression, risk and subgroup analyses showed that the precision of estimations improved in recent studies mostly due to increased sample sizes.

**Conclusions:**

Our cumulative meta-analysis would confirm that multiple aspects of social relationships are associated with cognitive decline. Yet, there is still evidence of publication bias and relevant information on study design is often missing, which could lead to an over-estimation of their statistical effects.

**Supplementary Information:**

The online version contains supplementary material available at (10.1186/s12889-022-12567-5).

## Background

Population ageing and related cognitive decline are global issues implying increased costs for governments, communities, families and individuals [1]. The WHO estimates that around 55 million people have dementia worldwide, with nearly 10 million new cases every year. The total number of people with dementia and severe cognitive impairment is projected to reach 78 million in 2030 and 139 million in 2050 (see: https://www.who.int/news-room/fact-sheets/detail/dementia). Dementia is a progressive and severely disabling condition often requiring intensive formal and informal home and/or institutional care [2]. It also tends to cluster with other diseases increasing the risk of unplanned hospitalisation, longer in-hospital stay and re-admissions, as well as functional impairment [3, 4]. The WHO estimates that the total global societal cost of dementia was US $ 1.3 trillion in 2019 but these costs are expected to surpass US$ 2.8 trillion by 2030, with half of them attributed to informal care (see: https://www.who.int/news-room/fact-sheets/detail/dementia). Understanding prevention and protection mechanisms that can minimise the risk of dementia and postpone its onset in an ageing population is key to reduce pressure on health care systems and welfare institutions, as well as to improve the quality of life of both families and caregivers.

Although complex factors may lead to individual transitions from normal cognition to dementia, research has recently shown that lifestyle-related factors, such as physical inactivity, tobacco use, unhealthy diet and the harmful use of alcohol, as well as several cardiovascular and metabolic conditions, such as hypertension and diabetes, increase the risk of cognitive impairment [5]. Recent studies have suggested that this risk greatly depends on the social context in which individuals are embedded [6, 7]. The modulation function of social conditions includes the prominent role of social networks, i.e., direct and indirect contacts between individuals in which information, attitudes and norms are shared [8–10]. For instance, a recent study showed that the network size and density, as well as the presence of weak ties (i.e., social bridging), moderate the association between brain atrophy and cognitive function, while marriage/cohabitation (i.e., social bonding) moderate the association between perceived stress and cognitive function [11].

Therefore, complex social and psychological factors and their underlying biological mechanisms could affect the risk of dementia, as well as on its prevention and protection. One of the most convincing hypotheses is that social activity and social engagement of older adults may promote neuro-protection and compensation, including the beneficial effect of physical exercise on neuro-degeneration [12]. For instance, previous research has shown that high level of social engagement and larger social networks are associated with better glucose regulation in adults without diabetes and better diabetes self-management that reduce the risk of dementia, thereby indicating a possible pathway that connects social relationships and cognitive abilities [13].

Previous sociological research has identified various structural and functional aspects of social relationships that may have either direct or indirect effects on cognitive decline among older adults [14–17]. Structural aspects typically include the individual network size (e.g., the number of frequent contacts, including family members, friends and acquaintances) and social activity (e.g., voluntary work, participation to community organisations, social clubs, neighbourhood associations) [8]. Functional aspects of social relationships typically include sources of social support, including material help and emotional support [18], and subjective perception of social integration against loneliness and social isolation [19]. For instance, rich and diverse social relationships can allow individuals to access information instrumental for better prevention and protection [6, 20]. Social relationships can also convey material and emotional support that can increase the capabilities of individuals to face critical events and processes related to ageing [12, 21]. These aspects shape the ‘personal community’ [22] of older adults conveying material and emotional resources that typically follow ego-specific social and spatial stratification and segmentation [23–26]. Along these spatial and social fault lines, various ‘social foci’ exist that determine individual heterogeneity of opportunities and constraints [27, 28], with potentially important implications on cognitive processes. For instance, loneliness and objective and subjective social isolation have a detrimental impact on the mental and physical health of older adults, which exceeds that of smoking 15 cigarettes per day or obesity [29, 30].

While research on this intersection of social factors and cognitive decline is growing, findings are still controversial especially regarding the effect of different types of social relationships and the accuracy of estimations of causal relationships between social and cognitive factors [31]. In a meta-analysis including relevant longitudinal cohort studies published until 2012, Kuiper and colleagues [32] found that despite heterogeneity in study design and measures, multiple aspects of social relationships were associated with cognitive decline. However, due to various sources of possible bias in measurements and estimates, these statistical associations should be interpreted with caution. For instance, due to reverse causality between social and cognitive factors, the authors of the meta-analysis concluded that more careful study design was needed to assess findings more systematically and disentangle various sources of complexity.

Here, we first aimed to update the previous meta-analysis by extending it to all relevant publications from 2012 to 2020. Second, we performed a cumulative meta-analysis that allowed us to assess the temporal evolution of the statistical estimates performed in all studies, including those reported in the previous meta-analysis. This was key to provide a more informative picture of the robustness of measurements and methodologies used in this growing field of area (+80% of articles from 2012-2020 compared to the previous period). Improving methods and measurements also increases our capability of assessing causal relationships between social and non-social factors, thus improving the quality of research design and measurements. We also need to understand whether certain direct or indirect interventions on social factors could be effective to either postpone or reduce the effect of cognitive decline for the general public.

## Methods

This systematic review and meta-analysis was pre-registered and the review protocol can be accessed at https://www.crd.york.ac.uk/prospero/ (ID: CRD42019130667). Reporting followed the Preferred Reporting Items for Systematic Reviews and Meta-Analyses (PRISMA) 2020 updated guidelines [33].

### Systematic search and study selection

We performed a systematic database search from Scopus and Web of Science (WOS) on 12 February 2019 using the same keywords and search design of the previous systematic review for all publications from 2012 to 2018. On 29 December 2020, the search was extended to all publications until 2020 by using the same search strings (see Supplementary Material, Section 1).

A total of 16,502 entries were initially selected resulting in 10,460 unique articles. Two members of our team independently screened the titles and abstracts giving 175 eligible articles. Any disagreements were resolved in consensus meetings. Persistent disagreements were resolved by a final decision being made by two additional authors. Following criteria used in the previous systematic review [32], articles were included if they: (i) were peer reviewed; (ii) reported an association between social relationships measured at baseline and the follow-up in a quantitative way; (iii) included a longitudinal prospective cohort study design conducted on the general population. Only articles published in English, German or French were included. Studies considering dementia as outcome were excluded. Note that we considered studies relying on samples including subjects living independently and community-dwelling.

### Data collection, items, risk of bias

The same two authors involved in the screening, then independently extracted data used in the study, i.e., population characteristics, timing of follow-up, measurement of social relationships, measurement of cognitive functioning, statistical methods and results. Whenever possible, estimates adjusted for potential confounding factors were used for the meta-analysis. For the sake of consistency with the previous meta-analysis, we considered the following potential confounding factors: (1) age; (2) presence of depressive symptoms; (3) alcohol use; (4) education; (5) baseline cognition; and (6) physical functioning. This last included at least one or a combination of the following: (i) physical activity; (ii) functional disability; (iii) chronic diseases, such as traumatic brain injuries, cardiovascular disease or cerebrovascular accident.

The methodological quality of the included studies was assessed independently by the two reviewers who had screened all entries with the Quality of Prognosis Studies in Systematic Reviews (QUIPS) tool [34]. The QUIPS tool includes six domains of possible bias that should be considered whenever evaluating the validity and bias in prognostic factors, each presented with prompting items and considerations. As regards the participation domain, we considered whether series of participants were consecutive and if participation was adequate compared to the initial number of recruited individuals. We evaluated study attrition according to data completeness with reference to the outcome and lack of differences between sample and dropout. We then assessed the validity of methods and the completeness of data to measure social relationships and cognitive abilities. We also considered whether the assessment of cognitive abilities and social relationships of participants was performed separately by different interviewers. We included measurement and inclusion in the analysis of any relevant confounding factors. We also included an item about minimisation of reverse causality by assessing whether the analysis was adjusted for baseline cognitive function or subjects with cognitive impairments or dementia were excluded at baseline. Finally, the statistical analysis and reporting domain were assessed for the risk of over-fitting [35].

Disagreements were resolved in consensus meetings. Again, to make quality evaluation consistent with the previous meta-analysis, the reviewers adopted the same tool used in the previous systematic review [32].

### Statistical analysis

We performed a meta-analysis using a random effects model to estimate the pooled estimates. We used the DerSimonian and Laird method to estimate the between-study variance components [36]. We assessed statistical heterogeneity among studies by using the *Q*-test based on the chi-squared statistics and quantified the proportion of total variation contributed by between-study variance through the *I*^2^ statistic [37]. We combined all selected papers from this study with those used in the meta-analysis in the previous systematic review [32]. Additionally, we performed a cumulative meta-analysis to map any temporal changes in the magnitude and significance of estimates for the association between social relationships and cognitive decline.

We then followed the previous review and stratified our statistical analysis by considering three categories of social relationships: (i) structural aspects; (ii) functional aspects; (iii) a combination of the two. Structural aspects of social relationships refer to the structure of social networks and social activities, such as the size, frequency and heterogeneity of social contacts [38–41]. Functional aspects of social relationships refer to sources support and social integration [42–45]. Finally, the combination between the two included composite indices of social network characteristics, social capital and social engagement [46, 47]. Whenever social relationship factors were given as categorical variables, we dichotomized them so that the lowest category (poor social relationships) was tested against the other categories combined. We then used the odds ratio based on the new two-by-two table [32].

We used odds ratios to represent the risk of developing cognitive impairment among people with poor social relationships compared to people with better social relationships. We interpreted hazard ratios as odds ratios. Given that studies mostly reported results with standardized and un-standardized coefficients from linear regression models, we converted these to odds ratios, as suggested by previous research [48]. Whenever in the original article any information for calculation of odds ratios and 95% confidence intervals were missing, we contacted the authors for any additional information.

When multiple articles were based on the same database, we selected results based on the following criteria: (i) an estimate for the meta-analysis; (ii) determinant measured as a composite measure of social relationship factors, or most compatible with the other studies; (iii) outcome measures such as global cognitive functioning, or most compatible with the other studies; (iv) longest follow-up duration; and (v) largest sample size.

We examined the heterogeneity sources by conducting stratified analyses for structural, functional and combined factors. We included the following characteristics: (i) year of publication (before 2006, 2007-2011, 2012-2018, after 2019); (ii) inclusion in the previous review [32]; (iii) geographic area (i.e., Asia, Europe, America); (iv) sample size (≤687, 688-1635, 1636-3413, >3413); (v) follow-up duration (≤3, 4-5, 6-9, >9); (vi) average age of baseline participants (≤65, 66-74, ≥75); (vii) outcome (i.e., cognitive function, cognitive decline); (viii) type of outcome (i.e., continuous, dichotomous), as reported in each study; (ix) social relationship measurement (i.e., low social activity, small social network size); (x) selected confounding factors (i.e., age, depression, alcohol consumption and physical activity) adjusted for.

We assessed publication bias by visual inspection of funnel-plots for asymmetry and through the Egger’s test for asymmetry [49].

We performed all the analyses through R version 4.1.2, using the “metafor” package [50]. Code is available in the Supplementary Material (Section 5).

## Results

### Study selection

**Fig. 1:**
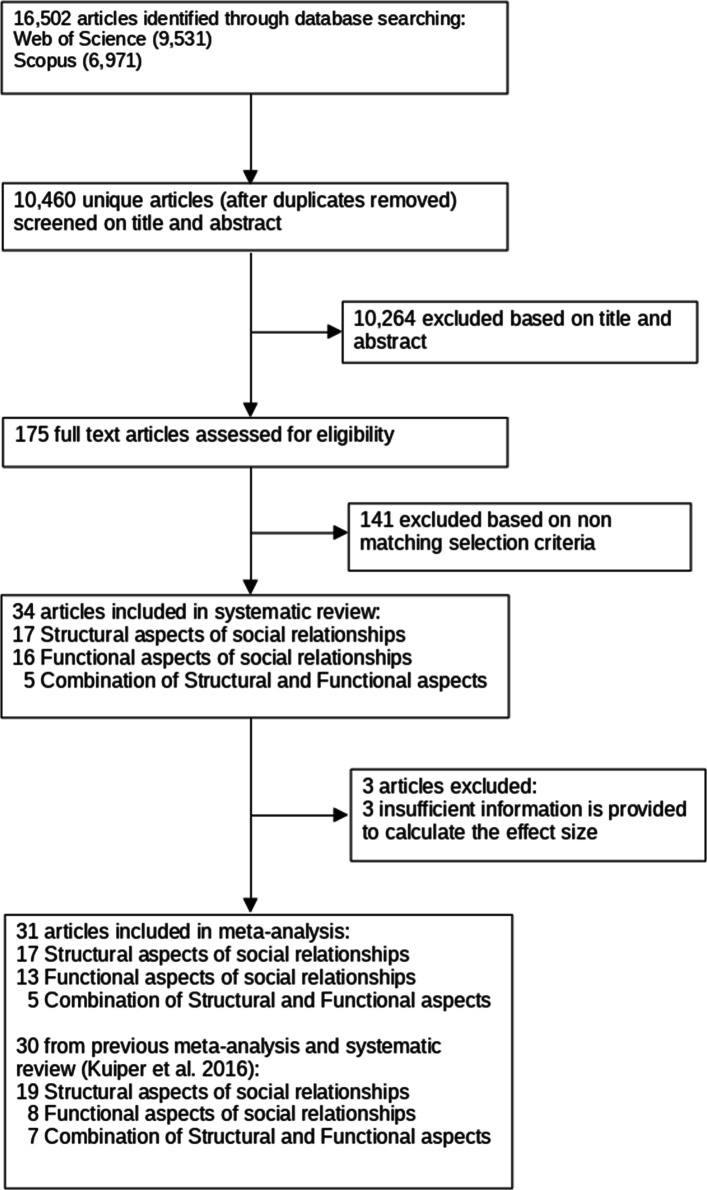
Selection flowchart. Selection flowchart for papers included in the systematic review

We selected a total of 34 unique publications according to our inclusion criteria (Fig. 1). Authors of four of these investigated more than one aspect of social relationships, resulting in 17 articles for structural aspects, 16 articles for functional aspects and 5 articles for combination of both (for detail on study characteristics of these 34 articles, see Supplementary Material, Table 1). Of the total 34 articles included in the systematic review, we included 31 in the meta-analysis.

**Fig. 2:**
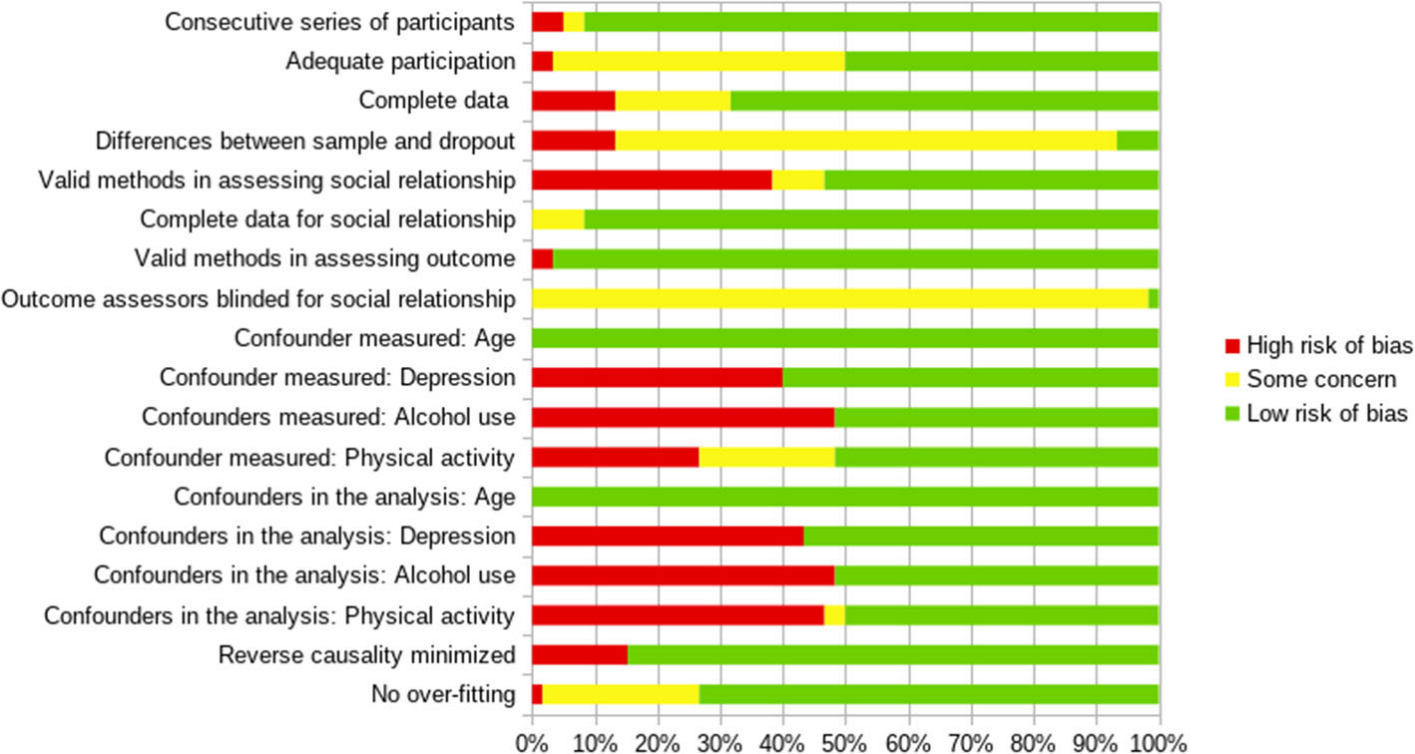
Quality bias. Quality bias analysis for articles included in the systematic review

Figure 2 shows a risk analysis for bias in all included studies using the QUIPS tool, as suggested by the Cochrane Prognosis Methods Group [35]. Results indicated that assessing adequate participation rate as a percentage of contacted individuals was impossible for 47% of articles due to unknown information. Most studies relied on large representative national surveys for which specific information was difficult to retrieve in dedicated websites or reports. We estimated that 93% of studies were prone to a risk of bias regarding differences between the final sample and dropouts, for which information was mostly unreported. The methods used in these studies to assess social relationships (38% of entries with insufficient methods) and cognitive decline (3% of entries with insufficient methods) were valid and consistent with previous literature. However, 98% of studies did not report whether outcome assessors were blinded with respect to social relationship factors. This raises a relevant methodological issue given that without this information, addressing the problem of over- or under-estimation is impossible.

We found that the confounding factors mostly included in these studies were: age, depression, alcohol use and physical activity. Among these, age was the only confounding factor measured and included in all studies. The other variables were measured and were only included in about 50% of studies. We found that 15% of studies presented a high risk of bias for reverse causality. Finally, 25% of studies present some risk of over-fitting (i.e., minimum of 10 participants in the smallest group per predictor and outcome variable).

### Synthesis of results: association

In this section, following [32]’s meta-analysis, we present findings by structural and functional aspects of social relationships, as well as on their combination.

#### Structural aspects of social relationships

In total, in 17 selected articles, authors examined the association between structural aspects of social relationships and cognitive decline [6, 14–17, 20, 38–41, 51–57]. Aspects included: social activity (i.e., participation to social clubs, community/religious organisations, voluntary work, etc., 21 articles), network size (i.e., number of frequent contacts, 6 articles) and social engagement/disengagement (i.e., composite indices of social activity and network size, 3 articles).

**Fig. 3:**
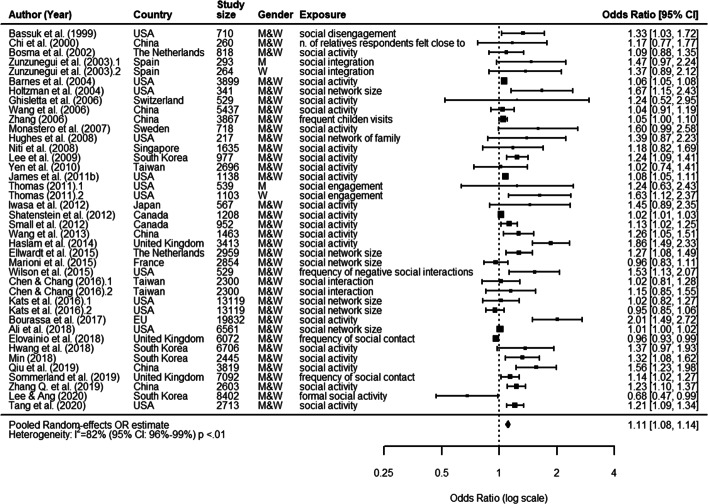
Structural aspects. Forest plot of the effect of structural aspects of social relationships on cognitive decline

In these studies, participants were on average 67.7 years old at baseline (range: 45 to 82), 50.8% were women. The average study duration was 11.0 years (range: 2.4 to 26 years), while the average sample size of the cohorts was 5,672 (range: 529 to 19,832). We found that both duration and sample size had substantially increased compared to studies included in the previous systematic review [32]. Since 2012, studies have mostly been performed using databases from United States (4), United Kingdom (3), South Korea (3) and China (3), so consolidating the existing *corpus* of studies. Finally, reported estimates from selected articles were characterised by smaller confidence intervals, probably due to the increasing use of larger samples rather than better measurements (see Fig. 3, which includes studies from both the previous meta-analysis [32] and our systematic review; for a complete description, see the Supplementary Material, Table 1).

The pooled random effects OR was estimated at 1.11 (95% CI: 1.08, 1.14). This confirmed previous findings and would indicate that structural aspects of social relationships are associated with cognitive decline. However, results were highly heterogeneous (*I*^2^= 82%, P <0.01 from *Q*-test), thus requiring a sub-group analysis to be performed.

Our sub-group analysis on sources of heterogeneity indicated that varying characteristics of the study had probably affected the presence and magnitude of an association between poor structural social relationships and cognitive decline. We found that considerable levels of heterogeneity (*I*^2^> 75%) could partially be explained by the following characteristics of the study: i) being published after 2007; ii) not being included in [32]; iii) relying on data from Europe or America; iv) relying on a sample size >687, ≤1635 and >3413; v) presenting a study follow-up greater than 5 years; vi) age of study participants (i.e., ≤65;66<*x*≤74;≥75); vii) reporting a continuous vs. dichotomized outcome (see Supplementary Material,Table 4).

**Fig. 4:**
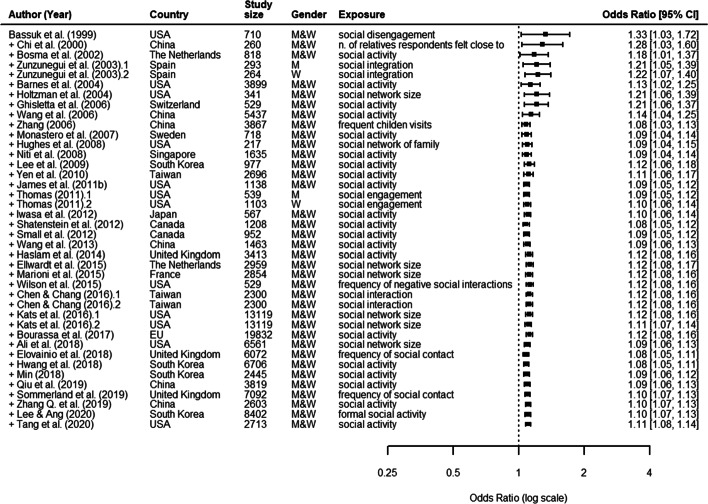
Structural aspects Cumulative meta-analysis forest plot of structural aspects of social relationships as predictors of cognitive decline

Figure 4 shows the results of a cumulative meta-analysis of the effect of structural aspects of social relationships on cognitive decline. Compared with studies included in the previous meta-analysis, we found more stable and precise estimates confirmed by narrowest confidence intervals. This trend can be traced back to studies since 2006.

#### Functional aspects of social relationships

In total, authors from 16 selected articles examined the association between functional aspects of social relationships and cognitive decline [16, 38, 39, 42, 43, 45, 47, 58–66]. Aspects included: social support (i.e., the availability of sources of material or emotional help, 12 articles), loneliness or isolation (i.e., subjective perception of loneliness or depression, 10 articles).

**Fig. 5:**
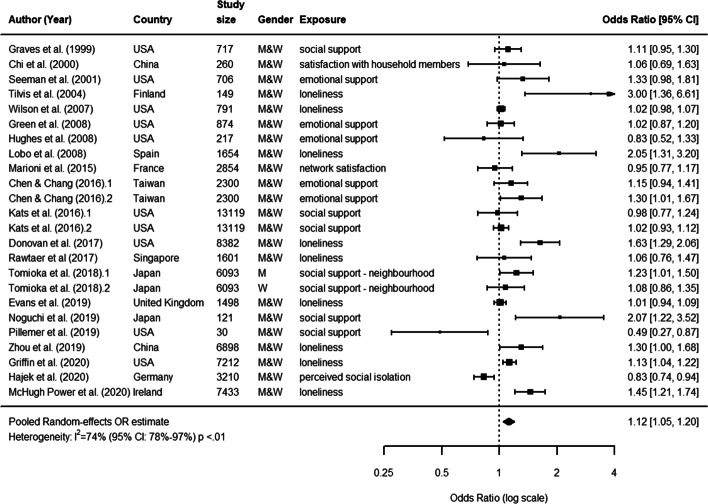
Functional aspects. Forest plot of the effect of functional aspects of social relationships on cognitive decline

In these cases, participants were on average 72.5 years old at baseline (range: 57 to 86), women were 55.8% of the total baseline samples, the average study duration was 7.5 years (range: 1.5 to 20), while the average sample size of cohorts was 4,192 (range: 121 to 13,119). Furthermore, both the duration and sample size substantially increased compared to studies included previously [32]. Since 2012, studies were mostly performed using databases from United States (4) and Japan (3). The remaining studies were from other countries of South-East Asia (3) and Europe (4) (see Fig. 5, which includes studies from both the previous meta-analysis [32] and our systematic review; for a complete description, see the Supplementary Material, Table 2).

The pooled random effects OR was estimated at 1.12 (95% CI: 1.05, 1.20). This confirmed previous findings and indicated that functional aspects of social relationships are associated with cognitive decline. However, results were again very heterogeneous (*I*^2^= 74%, *P*< 0.01 from *Q*-test), thus requiring a subgroup analysis to be performed.

Our sub-group analysis on sources of heterogeneity indicated that varying pre-specified characteristics had affected the presence and magnitude of an association between poor functional aspects of social relationships and cognitive decline. We found that considerable levels of heterogeneity (*I*^2^> 75%) could partially be explained by the following characteristics of the study: i) being published after 2019; ii) not being included in [32]; iii) relying on data from Europe; iv) relying on a sample size smaller than 687, or > 1635 and <= 3413. v) reporting a study follow-up with less than 3 and 5 years; vi) age of study participants lower than 65; vii) reporting a continuous vs. dichotomous outcome; viii) reporting cognitive function instead of cognitive decline as outcome; ix) reporting loneliness as social relationship measurement (see Supplementary material, Table 5).

**Fig. 6:**
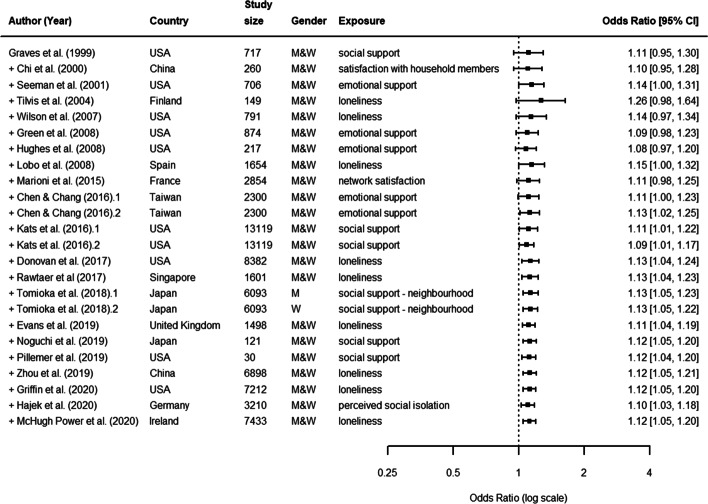
Functional aspects. Cumulative meta-analysis forest plot of functional aspects of social relationships as predictors of cognitive decline

Figure 6 shows the results of a cumulative meta-analysis of the effect of structural aspects on cognitive decline. As in the previous case, more recent studies showed more stable and precise estimates confirmed by narrowest confidence intervals and the departing from 1 in the odds ratio scale value.

#### Combination

**Fig. 7:**
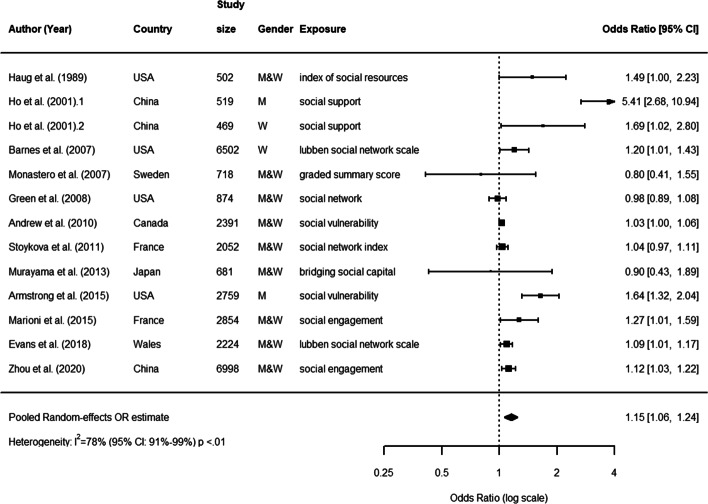
Combination aspects. Forest plot of the effect of a combination of functional and structural aspects of social relationships on cognitive decline

In total, authors from 5 selected articles examined the association between structural and functional aspects combined and cognitive decline [38, 44, 46, 67, 68]. In these cases, participants were on average 76.6 years old at baseline (range: 72 to 81), women were 40.5% of the total baseline samples. However, this under-representation of women in studies on a combination of aspects was driven by only one specific study [67], which focused on a male sample. The average study duration was 6.8 years (range: 2 to 20), while the average sample size of cohorts was 3103 (range: 681 to 6998). As in previous cases, both the duration and size had substantially increased compared to studies included in the previous systematic review [32]. Two of these studies relied on databases from China, while the others relied on databases from France, Sweden and USA (see Fig. 7, which includes studies from both the previous meta-analysis [32] and our systematic review; for a complete description, see Supplementary Material, Table 3).

The pooled random effects OR was higher than estimates of structural and functional aspects separated, though with a larger confidence interval (OR: 1.15, 95% CI: 1.01, 1.24). This indicates that structural and functional aspects of social relationships are associated with cognitive decline also when combined. However, again, results were very heterogeneous (*I*^2^= 74%, *P*< 0.01 from *Q*-test), thus requiring a sub-group analysis to be performed.

We found that considerable levels of heterogeneity (*I*^2^ 75%) could partially be explained by the following characteristics of the study: i) being published before 2006 or between 2012 and 2018; ii) being included in [32]; iii) relying on data from USA; iv) relying on a sample size <687, >1635 and ≤3413; iv) reporting a follow-up lower than 3 years; v) age of study participants higher than 75; vi) reporting a dichotomous vs. a continuous outcome; vii) reporting social support as social relationship measurement (see Supplementary Material, Table 6).

**Fig. 8:**
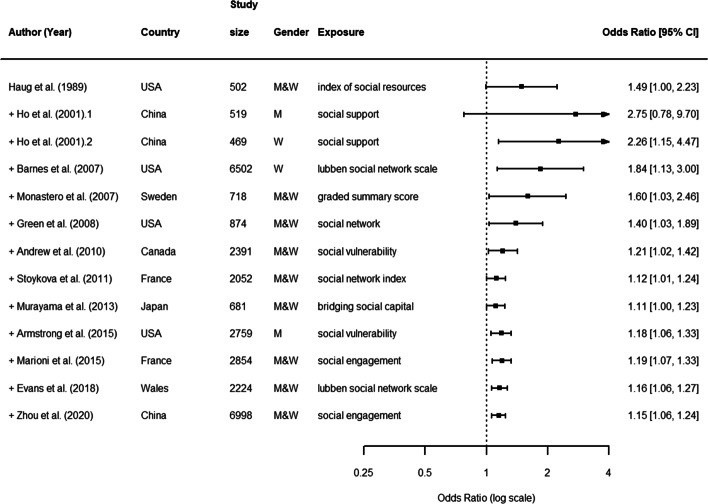
Combination aspects. Cumulative meta-analysis forest plot of a combination of functional and structural aspects of social relationships as predictors of cognitive decline

Figure 8 shows the results of a cumulative meta-analysis. As in previous cases, more recent studies showed more stable and precise estimates confirmed by narrowest confidence intervals and a reduction of the odds ratio.

### Robustness check and risk of bias

We examined the likelihood that our results, especially those on the structural aspects of social relationships, could be over-estimated due to publication bias in favour of positive effects. Our funnel plots for all three aspects of social relationships showed asymmetry and Egger’s test P values were <0.05 (see Supplementary Material,Figures 1-3). When considering only studies published more recently, not included in the original systematic review, we found a clearer asymmetry for structural aspects (*p* <0.01), while functional aspects and their combination (*p* =0.54 and 0.55, respectively) did not show any evidence of asymmetry. This would indicate that recent studies were more keen to include non-significant results than previously published research.

## Discussion

### Summary

Research on the association between social relationships and cognitive decline in older adults has recently increased in terms of numbers of publications, as well as qualitatively with larger and more international samples. Our results confirmed the effect of structural and functional aspects, as well as of their combination for cognitive decline: consistent with the previous meta-review [32], poorer social relationships predicted cognitive decline.

However, our results confirmed that there was still a considerable level of heterogeneity in the estimation of these statistical effects. After carefully examining this heterogeneity via sub-group analysis, we found that the most probable root-causes were certain methodological differences in social and cognitive variable measurements, the geographic characteristics of sampled populations and the duration of the follow-up study.

By means of a cumulative meta-analysis, we found that the precision and accuracy of estimations increased with a progressive reduction of 95% confidence intervals for all aspects of social relationships. However, this could be due to the increased sample sizes rather than precise variable measurements. Indeed, while studies before 2013 were based on data from local experimental design, authors of studies performed after 2013 increasingly relied on larger representative national surveys. Furthermore, note that 50% of studies did not control for alcohol use, about 40% for depression and about 50% for physical activity, thus not sufficiently controlling for relevant confounding factors. This may have led to an over-estimation of the association, which should be assessed in future reviews.

Additionally, we found other important methodological novelties in more recent studies. In some studies, structural equation modelling was used rather than regression analyses to test linear causal relationships among variables, thus simultaneously accounting for measurement errors [15, 64, 65]. This modelling technique is key to estimate causal mechanisms more accurately, especially in contexts in which reverse causality and varying possible causal paths need to be jointly assessed. Our test suggests that reverse causality between social relationships and cognitive decline has also been more carefully assessed in the most updated research. However, besides using more analytical statistical models, future research should also try to follow more robust sampling selection procedures, e.g., excluding participants with cognitive impairment or dementia at baseline. This would help minimising reverse causality issues and improve the robustness of results.

### Strengths and limitations

Our study has several strengths, which should be underlined. First, we extended and updated a previous systematic review [32], thus consolidating the systematic review approach in an interdisciplinary area where social scientists, geriatrics, neuro-epidemiologists and other experts are increasingly collaborating. Second, while the previous review addressed the study of cognitive decline and ageing on social relationships [32], our review presented a cumulative meta-analysis on the entire field, revealing that studies have achieved better OR estimates and progressively reduced the 95% confidence intervals for all aspects of social relationships, probably due to increased sample sizes.

Another important point is that our review included a bias risk analysis showing that methodological problems of these studies concern especially the weak control on certain confounding factors, including alcohol use, depression and physical activity, as well as on the lack of important detail on the participation rate and outcome assessors. These recurrent deficiencies must be solved in future research in order to improve the quality of findings assessment and stimulate cumulativeness and systematic comparisons.

As in the previous review, we had to face certain methodological challenges. On the one hand, we confirmed the significant heterogeneity between studies previously reported [32], which required meta-regression and subgroup analysis. On the other hand, we still detected possible publication bias for all three aspects of social relationships, which led us to conclude that estimates may well have been over-estimated. The prospective registration of observational studies and initiatives by journals and associations to increase data sharing and open data, occurring now in many other research areas [69], could improve methodological standards and quality of study design in this field.

Understanding potential mechanisms responsible for the complex associations between social relationships and cognitive decline requires to tackle complex pathways [70, 71]. On the one hand, social relationships can be instrumental for accessing relevant information for prevention and protection, stimulating intellectual and social engagement, increasing well-being and avoiding social isolation [31, 72, 73]. Indeed, social relationships express the full spectrum of lifestyles, including behaviours and norms that can lead to healthy or unhealthy outcomes [8]. For instance, recent research on the development of chronic diseases has shown that richer social networks can lower the speed of disease by improving prevention and protection [74]. Given that the chronic diseases is often associated with dementia incident [75], it is probable that this could be one pathway connecting social networks to cognitive decline. On the other hand, social networks convey a variety of emotional and material resources to individuals, whose complementarity or substitution effects are often difficult to estimate [31].

Besides certain interesting recommendations from the previous review [32], we suggest focusing on a ‘complexity hypothesis’. This is because social relationships are part of a complex social infrastructure linking individuals to a potential set of material and emotional resources related to cognition. It is likely that conventional measurements of social networks, such as network size and frequency of contacts, only partially reflect the complexity of personal networks. For instance, with data from the Longitudinal Aging Study Amsterdam (LASA), including 2,959 Dutch participants aged 54 to 85 at baseline in 1992 and six follow-ups covering a time span of twenty years, [20] showed that a reduction in network complexity was detrimental for cognitive functioning, neither explained by size of the network nor by simple presence of specific relationship type. The importance of non-redundant ties and the variety and heterogeneity of contacts [27] on health has been highlighted in a variety of studies [76, 77]. Being connected with non-redundant ties, all possibly with different information, skills and lifestyles, could be instrumental for older adults to access a greater variety of cognitive stimuli, obtain relevant information and achieve help and support [78, 79].

This is also linked to the so-called “focus theory”, which postulates that social relationships are more likely to form between individuals sharing certain ‘focused activities’ (e.g., community life, voluntary organisations) [26]. Given that ageing implies transitions and changes (e.g., retirement, widowhood and informal caregiving), we should expect that new ‘focused activities’ in later life could greatly affect egocentric networks of older adults imposing important re-configurations [80, 81]. As correctly suggested by [26], more careful attention to changes in network boundaries during later life and the inclusion of space as a social environment where most of these ‘focused activities’ take place, could improve our understanding of the effect of these network changes on cognitive decline of older adults [26].

## Conclusions and further research

This review has updated a previous meta-analysis on the effect of social relationships on cognitive decline in longitudinal cohort studies performed in 2016. We confirmed previous evidence on the importance of multiple aspects of poor social relationships, including structural, functional and a combination of these factors, to predict cognitive decline. Our cumulative meta-analysis would indicate that the precision of estimations has increased, at least since 2006, probably due to the increased sample sizes of these studies. However, deficiencies and problems persist, especially in study design (e.g., missing information on dropouts) and measurements.

Our results suggest that future research should consider the complexity of social factors associated with cognitive decline more carefully by improving measurements, especially reconstructing personal networks with data on alters’ alters so that the variety and heterogeneity of contacts can be estimated more precisely, including network boundaries and redundancy [26]. While this can be difficult for longitudinal cohort studies, less conservative and more explorative studies on new, ad-hoc samples, including experimental research, could help to test the accuracy of more complex network measurements, thus providing insights on how to incorporate these measurements in longitudinal cohort studies.

Finally, there is a need for research exploring the interplay between social networks, chronic diseases in adult life and cognitive decline in older age. This could also improve our understanding of the possible short and long-term impacts of the COVID-19 pandemic on new clinical conditions. The COVID-19 pandemic has hit especially hardly people in lower socio-economic strata of the population. Given that its consequences will probably increase health inequalities [4], we need more information on how individuals react to these changing social conditions and have adapted to external shocks [82].

## Supplementary Information

**Additional file 1** *supplementary-material.pdf* includes:
• search strings for the selection of papers from Scopus and Web of Science databases
• a table summing up characteristics of articles included in the systematic review
• funnel plots of structural, functional and combination of aspects of social relationships
• tables of subgroup analysis
• code for the analysis

**Additional file 2** *supp-mat-replication.csv* includes data for meta-analysis replication.

## Data Availability

Data and code to replicate the meta-analysis are included as supplementary information. The dataset used for the systematic review has been licensed for the current study from the German Competence Centre for Bibliometrics (Kompetenzzentrum Bibliometrie: https://bibliometrie.info/), and so is not publicly available. Data are however available from the authors upon reasonable request and with permission of German Competence Centre for Bibliometrics.
