# Supplementary material for: The effect of social relationships on cognitive decline in older adults: an updated systematic review and meta-analysis of longitudinal cohort studies
Source: BMC Public Health. 2022 Feb 11;22:278. doi: 10.1186/s12889-022-12567-5 (PMC8831686; doi:10.1186/s12889-022-12567-5)
Supplement: Supplementary file 1 — Additional file 1 supplementary-material.pdf includes: • search strings for the selection of papers from Scopus and Web of Science databases • a table summing up characteristics of articles included in the systematic review • funnel plots of structural, functional and combination of aspects of social relationships • tables of subgroup analysis • code for the analysis [file 12889_2022_12567_MOESM1_ESM.pdf]

Supplementary Material for “The Effect of Social Relationships on Cognitive Decline in Older Adults: An Updated Systematic Review and Meta-Analysis of Longitudinal Cohort Studies”

1. Search strings

Find all ”article” OR ”review” publications ”title” OR ”abstract” OR ”keywords” of the publication includes a lower- OR title-case version of these word pairs

[”Cognitive Function” OR ”Cognitive Decline” OR ”Cognitive Deficit” OR ”Cognitive Impairment” OR ”Cognition Loss” OR ”Cognitive Loss” OR ”Cognitive Abilit” OR ”Dement” OR ”Alzheimer” OR ”Cognition” OR ”Cognitive Status” OR ”Cognitive Change” OR ”Cognition Change” OR ”Cognitive Performance” OR ”Cognitive Disfunction”]

AND

[”Loneliness” OR ”Social Support” OR ”Social Isolation” OR ”Social Participation” OR ”Social Engagement” OR ”Social Disengagement” OR ”Social Integration” OR ”Personal Network” OR ”Social Network” OR ”Social Activit” OR ”Social Tie” OR ”Social Relation” OR ”Social Interaction” OR ”Social Withdrawal” OR ”Social Capital” OR ”Social Contact” OR ”Social Embeddedness” OR ”Family Relation” OR ”Kinship Relation” OR ”Friendship” OR ”Social Influence” OR ”Social Vulnerability”]

2. Supplementary Tables

Table 1: Structural aspects of social relationship.

Study characteristics				Population characteristics			Adjustment for covariates	Outcome	Social relationship assessment	Results	
Author	Country	Study duration (yrs)	*N* in the analysis	In- and exclusion criteria	Age mean (SD), range (yrs)	Women (%)				Original from paper	OR (95% CI) in meta-analysis
Ali et al (2018)	USA	19	6561	Inclusion: Resident in one of 3 adjacent selected neihgborhoods in South Side Chicago; 65+ years old	74 (7.0), 61-108	62	Gender, self-reported race, lifetime SES (childhood SES, education, occupational status, current income), marital status, number of medical conditions, physical function summary score, cognitive function summary score, network size and diversity mutual adjustment	Cognitive function. Continuous, assessed with immediate and delayed recall East Boston Story oral Symbol Digit Modalities Test and MMSE.	Structural: Social network size. Continuous, based on number of children, other relatives, friends and neighbours. Network size cut at 12, then summed across network type. Range 0-48.	Unstandardized Beta (SE, *p*-value): 0.004 (0.001, p<0.001)	1.01 (1.00, 1.02)

Continues in the following page

Continuing from the previous page

Study characteristics				Population characteristics			Adjustment for covariates	Outcome	Social relationship assessment	Results	
Author	Country	Study duration (yrs)	*N* in the analysis	In- and exclusion criteria	Age mean (SD), range (yrs)	Women (%)				Original from paper	OR (95% CI) in meta-analysis
Ali et al (2018)									Structural: Social network diversity. Measured with network diversity index. Range (1-4). Increasing score indicate increasing diversity in social relationships inside the network.	Unstandardized Beta (SE, *p*-value): 0.109 (0.013, p<0.001)	1.55 (1.4, 1.73)
Bourassa et al (2017)	EU	6	19832	Inclusion: Participating in SHARE; aged 50+ at baseline. Exclusion: Present in only one wave (baseline or follow-up); same household of other primary respondents	64.4 (10.1), 62-82	52.7	Age at baseline, gender, income, depressive symptoms, physical health, physical activity level.	Cognitive function. Continuous, assessed with executive function task (category verbal fluency).	Structural: Social activity. Continuous, assessed using a sum score of 4-category social activity (participation to: voluntary or charity work; sport, social, or other kind of club; religious organization; political or community organization). Higher scores indicate higher levels of social activity.	Standardized Beta (95% CI): 0.19 (0.14,0.23), *p* <0.01.	2.01 (1.49, 2.72)

Continues in the following page

Continuing from the previous page

Study characteristics				Population characteristics			Adjustment for covariates	Outcome	Social relationship assessment	Results	
Author	Country	Study duration (yrs)	*N* in the analysis	In- and exclusion criteria	Age mean (SD), range (yrs)	Women (%)				Original from paper	OR (95% CI) in meta-analysis
Chen and Chang (2016)	Taiwan	14	2300	Inclusion: Participating in the Taiwan Longitudinal Study on Aging in 1989; at least two measures of SPMSQ during 1993 to 2007. Exclusion: Younger than 65 years-old in 1993; died before 1993; incorrect information on death; stroke diagnosis in 1993	70.9 (5.0), 65+	44.8	Age, sex, education, health status (BMI, chronic diseases, depression symptom score), health behaviour (smoking, alcohol drinking, physical activity), physical function (activities of daily living, instrumental activities of daily living and mobility tasks).	Cognitive function. Continuous, assessed with five item SPMSQ, validated by a Chinese version of MMSE. Score ranging 0 to 5, higher score indicates higher cognitive functioning.	Structural: Social interaction. Measured with items investigating involvement in playing games and socializing with friends, neighbours and relatives. Higher score indicates higher interaction	Starting high and declining OR (95% CI): 0.98 (0.78, 1.23)	1.02 (0.81, 1.28)
Chen and Chang (2016)										Starting low and declining OR (95% CI): 0.87 (0.64, 1.17)	1.15 (0.85, 1.55)

Continues in the following page

Continuing from the previous page

Study characteristics				Population characteristics			Adjustment for covariates	Outcome	Social relationship assessment	Results	
Author	Country	Study duration (yrs)	*N* in the analysis	In- and exclusion criteria	Age mean (SD), range (yrs)	Women (%)				Original from paper	OR (95% CI) in meta-analysis
Ellwardt et al (2015)	The Netherlands	6.2 (5.8), max 19.8	2959	Inclusion: Participating in the Longitudinal Aging Study Amsterdam (LASA); born between 1908-1937; living in areas in and around cities of Zwolle, Oss, and Amsterdam	73.9 (8.5), 54-100	51.5	Age, gender, education, living with partner, physical functioning, time	Cognitive function. Continuous, assessed with MMSE (range 0-30)	Structural: Social network size. Measured as total count of all members in the personal network	Unstandardized Beta (95% CI): 0.021 (0.013,0.028), p <0.001	1.27 (1.08, 1.48)
Ellwardt et al (2015)									Structural: Social network complexity. Measured with the Cohen's Social Network Index, based on the number of social roles the respondent has regular contact with.	Unstandardized Beta (95% CI): 0.110 (0.075,0.144), $p < 0.001$	1.3 (1.1, 1.53)

Continues in the following page

Continuing from the previous page

Study characteristics				Population characteristics			Adjustment for covariates	Outcome	Social relationship assessment	Results	
Author	Country	Study duration (yrs)	*N* in the analysis	In- and exclusion criteria	Age mean (SD), range (yrs)	Women (%)				Original from paper	OR (95% CI) in meta-analysis
Elovainio et al (2017)	UK	26	6072	Inclusion: 35-55 years old in 1985-88; participating in the Whitehall II Study	61 (0.1)	29	Age, sex, ethnicity, socioeconomic status (occupation), level of C-reactive protein (CRP), blood pressure, low-density lipoprotein cholesterol, fasting glucose, drinking, BMI	Cognitive decline. Continuous, assessed with the Alice Heim 4-I scale, short-term verbal memory assessment, verbal fluency test. Single test scores were combine in standardized global *z*-score. Measured at baseline and follow-up. Three-trajectory (low, medium, high) of global cognitive function derived with GBTM methods.	Structural: Frequency of social contact. Continuous, measured with the Berkman/Syme social network index.	RRR (95% CI) Low vs high: 0.96 (0.93,0.99)	0.96 (0.93, 0.99)

Continues in the following page

Continuing from the previous page

Study characteristics				Population characteristics			Adjustment for covariates	Outcome	Social relationship assessment	Results	
Author	Country	Study duration (yrs)	N in the analysis	In- and exclusion criteria	Age mean (SD), range (yrs)	Women (%)				Original from paper	OR (95% CI) in meta-analysis
Haslam et al (2014)	UK	4	3413	Inclusion: Born before March 1952; take part in wave 3, 4 and 5 of English Longitudinal Study on Ageing; complete information on key variables	62.6 (8.9), 50-99	57.3	Age, sex, socioeconomic status (income decile), physical health	Cognitive function. Continuous, computed for each wave using factor analysis on five items: orientation in time and space (assessed with MMSE), immediate and delayed memory (assessed with tasks from the Health and Retirement Study), verbal fluency, prospective memory (assessed with tasks from the Medical Research Council Cognitive Function and Ageing Study)	Structural: Social activity. Continuous, computed with factor analysis. The "social activity" factor comprises measures that indexed societal and civic engagement, participation in cultural activities and number of group memberships	Standardized Beta (95% CI): 0.17 (0.11, 0.23), $p < 0.001$	1.86 (1.49, 2.33)

Continues in the following page

Continuing from the previous page

Study characteristics				Population characteristics			Adjustment for covariates	Outcome	Social relationship assessment	Results	
Author	Country	Study duration (yrs)	*N* in the analysis	In- and exclusion criteria	Age mean (SD), range (yrs)	Women (%)				Original from paper	OR (95% CI) in meta-analysis
Hwang et al (2018)	South Korea	8	6706	Inclusion: Participating in the KLOSA survey; normal cognitive function at baseline (K-MMSE>24). Exclusion: Missing covariates at baseline	58 (0.1)	50.1	Age, gender, education, household income quartile, working status, marital status, residence, physical activity, smoking, drinking, ADL, depression, comorbidity	Global cognitive functioning. Continuous, assessed with K-MMSE at baseline and follow-up	Structural: Social activity. Assessed at baseline using four separate dimension: participation in religious groups, social gatherings, Alumni, volunteer work	Religious groups Unstandardized Beta (SE, *p*-value): -0.01 (0.18, 0.96)	0.99 (0.71, 1.38)
Hwang et al (2018)										Social gatherings Unstandardized Beta (SE, *p*-value): 0.29 (0.16, 0.07)	1.37 (0.97, 1.92)
Hwang et al (2018)										Alumni Unstandardized Beta (SE, *p*-value): -0.16 (0.16, 0.34)	0.86 (0.62, 1.18)

Continues in the following page

Continuing from the previous page

Study characteristics				Population characteristics			Adjustment for covariates	Outcome	Social relationship assessment	Results	
Author	Country	Study duration (yrs)	*N* in the analysis	In- and exclusion criteria	Age mean (SD), range (yrs)	Women (%)				Original from paper	OR (95% CI) in meta-analysis
Hwang et al (2018)										Volunteer work Unstandardized Beta (SE, *p*-value): 0.17 (0.33, 0.60)	1.08 (0.80, 1.47)

Continues in the following page

Continuing from the previous page

Study characteristics				Population characteristics			Adjustment for covariates	Outcome	Social relationship assessment	Results	
Author	Country	Study duration (yrs)	*N* in the analysis	In- and exclusion criteria	Age mean (SD), range (yrs)	Women (%)				Original from paper	OR (95% CI) in meta-analysis
Kats et al (2016)	USA	20	13119	Inclusion: Participating to ARIC study (aged 45-64 in 1987-1989); living in Forsyth County, NC; Jackson, MS; suburbs of Minneapolis, MN and Washington County, MD. Exclusion: Not African American or Caucasian, African American in Minneapolis, African American in Washington County; missing global *z*-scores at baseline; missing education; missing ISEL-SF or LSNS	57 (5.7)	56.2	Age, sex, study centre, education, smoking, alcohol compsumption, hypertension, diabetes	Cognitive function. Continuous, measured with standardized global z-score of: Digit Symbol Sostitution Test (DSST-executive function, prcessing speed), Delayed Word Recall Test (DWRT- verbal learning, immediate memory) and Word Fluency Test (WFT - executive function, expressive language)	Structural: Social network size. Dichotomous, "low risk" vs "isolated/high risk". Measured using the Lubben Social Network Scale (LSNS). Total score ranges 0-50, dichotomized with cutoff at 31	African Americans *z*-score (95% CI): -0.01 (-0.11, 0.13), p =0.87	1.02 (0.82, 1.27)
Kats et al (2016)										Caucasian Americans *z*-score (95% CI): -0.03 (-0.09, 0.03), p =0.29	0.95 (0.85, 1.06)

Continues in the following page

Continuing from the previous page

Study characteristics				Population characteristics			Adjustment for covariates	Outcome	Social relationship assessment	Results	
Author	Country	Study duration (yrs)	*N* in the analysis	In- and exclusion criteria	Age mean (SD), range (yrs)	Women (%)				Original from paper	OR (95% CI) in meta-analysis
Lee and Ang (2020)	South Korea	6	8402	Inclusion: Enrolled in the Korean Longitudinal Study of Aging (KloSA). Exclusion: Aged below 60; cognitive impairment at baseline; unreliable values on cognitive impaiment score over waves; one or more variables of interest missing in all waves	66.5 (5.7), 60-91	44.5	Age, education, marital status, log total annual household income, rurality, living alone, heart-related diseases, ADL, depression, phisical activity	Cognitive impairment. Dichotomized, measured using the Korean version of MMSE. Cutoff at 24	Structural: Informal social activity. Dichotomous, assessed as time spent in meeting family and grandparenting (1 if participated in activity in last year, 0 if not)	OR (95% CI): 0.58 (n.a.), *p* <0.01	1.72 (1.18, 2.52)
Lee and Ang (2020)									Structural: Formal social activity. Dichotomous, assessed as participation in senior community centers, educational programs, alumni societies, volunteer, political groups (1 if participated in activity in last year, 0 if not)	OR (95% CI): 1.46 (n.a.), *p* <0.05	0.68 (0.47, 0.99)

Continues in the following page

Continuing from the previous page

Study characteristics				Population characteristics			Adjustment for covariates	Outcome	Social relationship assessment	Results	
Author	Country	Study duration (yrs)	*N* in the analysis	In- and exclusion criteria	Age mean (SD), range (yrs)	Women (%)				Original from paper	OR (95% CI) in meta-analysis
Marioni et al (2015)	France	20	2854	Inclusion: Participated in the Paquid database; 65+ at baseline (1988); residing at home at baseline. Exclusion: Partial or absent data	77 (6.8)	59	Age, gender, education (low, medium, high), marital status	Cognitive decline. Categorical (non-decliners, moderate decliners, fast decliners). Cognitive ability assessed using MMSE, verbal fluency (Isaac's Set Test truncated at 15), abstract thinking (Wechsler Similarities Test), episodic memory and learning (Wechsler Paired Associate Test), processing speed (Digit Symbol Substitution Test), and immediate visual memory (Benton Visual Retention-Test)	Structural: Social network size. Dichotomous, "large" (network $\geq$ 8 people) vs "small" (network $<$ 8 people)	HR (95% CI): 1.04 (0.90, 1.20)	0.96 (0.83, 1.11)

Continues in the following page

Continuing from the previous page

Study characteristics				Population characteristics			Adjustment for covariates	Outcome	Social relationship assessment	Results	
Author	Country	Study duration (yrs)	*N* in the analysis	In- and exclusion criteria	Age mean (SD), range (yrs)	Women (%)				Original from paper	OR (95% CI) in meta-analysis
Min (2018)	South Korea	6	2445	Inclusion: Korean resident in South Korea (no Juju Island); 60+ years old at baseline; K-MMSE 24+ at baseline; completed MMSE in 3 out of 4 waves	67.5 (5.6), 60-91	46	Age, gender, marital status, education, functional health (ADL, IADL), depression, regular exercise, smoking, drinking	Cognitive decline. Dichotomous, assessed with MMSE. Cutoff: MMSE<24	Structural: Social activity. Continuous, range: 0-6. Assessed with participation at church, social clubs, sports clubs, alumni societies, volunteer groups, political organizations	OR (95% CI): 0.76 (0.60,0.96), $p < .05$	1.32 (1.07, 1.61)
Qiu et al (2019)	China	16	3819	Inclusion:Participated in the Chinese Longitudinal Healthy Longevity Study (CLHLS) between 1998-2014; aged >79. Exclusion: Cerebrovascular disease; Parkinson diagnosis; totally limited physical function; extremely incomplete information	89.2 (6.8), 80+	48.2	Age, sex, marital status, occupation, smoking, drinking, vegetarian, diabetes, depression	Cognitive impairment. Dichotomized, measured using the Chinese revised version of MMSE. Cutoff point of <24	Structural: Social activity. Dichotomous. Frequency of taking part in social activities assessed with a 3-point scale (never, sometimes, almost every day), rescaled to “never” vs “almost every day”	HR (95% CI): 0.64 (0.50, 0.81)	1.56 (1.23, 1.99)

Continues in the following page

Continuing from the previous page

Study characteristics				Population characteristics			Adjustment for covariates	Outcome	Social relationship assessment	Results	
Author	Country	Study duration (yrs)	*N* in the analysis	In- and exclusion criteria	Age mean (SD), range (yrs)	Women (%)				Original from paper	OR (95% CI) in meta-analysis
Sommerlad et al (2019)	UK	14.3 (5.6)	7092	Inclusion: Civil servants in London; 35-55 years old at baseline; completed all 12 waves	44.9 (6.1)	33.1	Age, gender, ethnicity, education, SES (grade of last employment), employment status, marital status, smoking, drinking, physical activity	Global cognitive function. Continuous, assessed with global cognitive score (phonemic fluency + semantic fluency + short-term verbal memory + Alice Heim 4-I test of verbal and mathematical reasoning). Standardized z-scores	Structural: Frequency of social contact. Continuous, measured with Berkman/Syme social network index (range 0-16)	Unstandardized Beta (95% CI): -0.01 (-0.03, 0.01)	1.14 (1.02, 1.27)

Continues in the following page

Continuing from the previous page

Study characteristics				Population characteristics			Adjustment for covariates	Outcome	Social relationship assessment	Results	
Author	Country	Study duration (yrs)	N in the analysis	In- and exclusion criteria	Age mean (SD), range (yrs)	Women (%)				Original from paper	OR (95% CI) in meta-analysis
Wang et al (2013)	China	2.4	1463	Inclusion: Aged 65+; living in Sichuan or Shandong province. Exclusion: Having hearing problems or physical disabilities; missing follow-up visit; baseline global cognitive score in the bottom 10% of distribution	71 (5)	49.2	Age, gender, year of schooling, marital status, household composition, alcohol consumption, smoking, medical history and fracture, height, weight, BMI, APOE genotype	Cognitive function. Continuous, measured using the Community Screening Instrument for Dementia (CSID). Higher scores for higher cognitive function	Structural: Social activity. Dichotomous, assessed with frequencies of engagement in visiting family or friends, receiving visitors at home, giving advice. Final score has been dichotomized in “low activity” (lower tertile) and “high activity” (middle and upper tertile)	Standardized Beta (95% CI): 0.13 (-0.23, -0.03), p <0.05	1.26 (1.05, 1.51)

Continues in the following page

Continuing from the previous page

Study characteristics				Population characteristics			Adjustment for covariates	Outcome	Social relationship assessment	Results	
Author	Country	Study duration (yrs)	*N* in the analysis	In- and exclusion criteria	Age mean (SD), range (yrs)	Women (%)				Original from paper	OR (95% CI) in meta-analysis
Wilson et al (2015)	USA	10	529	Inclusion: Participating in Rush Memory and Aging Project; aged 50+; no diagnosis of dementia prior to enrollment; agreement to yearly clinical examination; brain autopsy at death. Exclusion: Dementia or MCI at baseline	81.4 (7.1), 50+	78.9	Age, sex, education, social network size, social activity, loneliness, depressive symptoms, ability to cope with stress, negative life events	Cognitive function. Continuous, standardized. Composite measure of global cognition based on 19 tests measuring: episodic memory, semantic memory, working memory, perceptual speed, visuospatial ability	Structural: Frequency of negative social interactions. Assessed with 12 items on four domains: neglect of rejection by others; others' unwanted intrusion or advice; failure by others to provide help; unsympathetic or insensitive behavior by others. Scores for the total scale obtained by averaging item scores	HR (95% CI): 1.53 (1.13, 2.07)	1.53 (1.13, 2.07)
Tang et al (2020)	USA	2.5	2713	Inclusion: Chinese older adults residing in Chicago, USA	72.8 (8.3), 59-103	57.9	Age, gender, education, income, self-rated health, years living in the neighborhood, years living in USA, marital status	Cognitive function. Continuous, assessed with C-MMSE	Structural: Social activity. Continuous, assessed as frequencies of participating in following activities: going out, visiting friends, inviting guests at home	Standardized Beta (95% CI, *p*-value): 0.10 (, *p* <0.001)	1.21 (1.09, 1.34)

Continues in the following page

Continuing from the previous page

Study characteristics				Population characteristics			Adjustment for covariates	Outcome	Social relationship assessment	Results	
Author	Country	Study duration (yrs)	*N* in the analysis	In- and exclusion criteria	Age mean (SD), range (yrs)	Women (%)				Original from paper	OR (95% CI) in meta-analysis
Zhang et al (2019a)	China	16	2603	Inclusion: Resident in one of 22 sample areas in 31 provinces in China; 80+ years old at baseline; 65-79 years old in 2002; 64 years old in 2005	n.a.	52.9	Age, gender, education, marital status, income, place of residence, psychological well-being, fresh fruit intake, fresh vegetable intake, smoking, drinking, exercising, outdoor activities, play cards/mahjong, ADL, hypertension, diabetes, cardiovascular disease, orthopedic disease	Cognitive impairment. Dichotomous, assessed with C-MMSE (range: 0-30), cut-off < 24	Structural: Social activity. Continuous, measured as frequency of engagement in social activities. Score ranges 1-5. Higher score indicate higher frequency of engagement in social activities	OR (SE, 95% CI, *p*-value): 0.81 (0.04; 0.73, 0.91; $p < 0.001$)	1.23 (1.11, 1.38)

Continues in the following page

Continuing from the previous page

Study characteristics				Population characteristics			Adjustment for covariates	Outcome	Social relationship assessment	Results	
Author	Country	Study duration (yrs)	*N* in the analysis	In- and exclusion criteria	Age mean (SD), range (yrs)	Women (%)				Original from paper	OR (95% CI) in meta-analysis
Zhang et al (2019b)	USA	2	2543	Inclusion: Chinese; resident in Greater Chicago area; able to provide informed consent; 60+ years old	75.5 (8.1)	58.3	Age, sex, education, marital status, annual income, ADL, IADL, comorbidity	Global Cognition. Continuous, assessed with: general mental status (C-MMSE), episodic memory (East Boston Memory Test), perceptual speed (11-item Symbol Digit Modalities Test), working memory (Digit Span Backward from Wechsel Memory Scale Revised). Average *z*-scores of 4 tests	Structural: Social activity. Continuous, measured with an index of participation into activities that are socially stimulating and ranging 0-32	Unstandardized Beta (95% CI), *p*-value: 0.005 (SE=0.00), p =0.04	

Concluding from the previous page

Table 2: Functional aspects of social relationships.

Study characteristics				Population characteristics			Adjustment for covariates	Outcome	Social relationship assessment	Results	
Author	Country	Study duration (yrs)	*N* in the analysis	In- and exclusion criteria	Age mean (SD), range (yrs)	Women (%)				Original from paper	OR (95% CI) in meta-analysis
Chen and Chang (2016)	Taiwan	14	2300	Inclusion: Participating in the Taiwan Longitudinal Study on Aging in 1989; at least two measures of SPMSQ during 1993 to 2007. Exclusion: Younger than 65 years-old in 1993; died before 1993; incorrect information on death; stroke diagnosis in 1993	70.9 (5.0), 65+	44.8	Age, sex, education, health status (BMI, chronic diseases, depression symptom score), health behaviour (smoking, alcohol drinking, physical activity), physical function (activities of daily living, instrumental activities of daily living and mobility tasks)	Cognitive function. Continuous, assessed with five item SPMSQ, validated by a Chinese version of MMSE. Score ranging 0 to 5, higher score indicates higher cognitive functioning	Functional: Emotional support. Assessed with 3 items investigating respondents being cared for when ill and being listened to by friends or relatives. Each item was scores 0 (no) or 1 (yes)	Starting high and declining	1.15 (0.94, 1.41)
Chen and Chang (2016)									Functional: Emotional support. Assessed with 3 items investigating respondents being cared for when ill and being listened to by friends or relatives. Each item was scores 0 (no) or 1 (yes)	Starting low and declining	1.30 (1.01, 1.67)

Continues in the following page

Continuing from the previous page

Study characteristics				Population characteristics			Adjustment for covariates	Outcome	Social relationship assessment	Results	
Author	Country	Study duration (yrs)	*N* in the analysis	In- and exclusion criteria	Age mean (SD), range (yrs)	Women (%)				Original from paper	OR (95% CI) in meta-analysis
Donovan et al (2017)	USA	12	8382	Inclusion: Participating in the Health and Retirement Study (HRS); 65+ in 2000; living in households in the contiguous US; being non-Hispanic white or black American	73.2 (6.5)	60	Age, sex, race, years of education, wealth, income, social network, health conditions and depression	Global cognitive score. Continuous, measured with Telephone Interview for Cognitive Status. Participants who were too impaired to undergo direct testing, proxy respondents rated the participant's memory using a 5-point Likert scale and completed the 16-item version of the Informant Questionnaire for Cognitive Decline (IQCODE)	Functional: Loneliness. Evaluated using CES-D scale (8-item version: felt depressed, felt everything was an effort, restless sleep, were happy, felt lonely, enjoyed life, felt sad, could not get going). Loneliness was rated as present if participants responded affirmatively to the loneliness question	Unstandardized Beta (95% CI): -0.2 (-0.3, -0.1), $p < 0.002$	1.63 (1.29, 2.06)

Continues in the following page

Continuing from the previous page

Study characteristics				Population characteristics			Adjustment for covariates	Outcome	Social relationship assessment	Results	
Author	Country	Study duration (yrs)	*N* in the analysis	In- and exclusion criteria	Age mean (SD), range (yrs)	Women (%)				Original from paper	OR (95% CI) in meta-analysis
Eisele et al (2012)	Germany	1.5	1869	Inclusion: 75+ yrs old; absence of dementia at baseline; regular patient of 1 of 138 primary care practices in 6 German cities; at least 1 consultation with general practitioner within last year. Exclusion: Residence in a nursing home; consultations by home visit only; severe illness fatal within 3 months (according to practitioner); insufficient ability to speak German; deafness, blindness and insufficient ability to consent	82.4 (3.3), 79-95	65.9	Age, gender, education, cognitive function at W1, sensory impairment, health status, physical activity, cardiovascular illness, alcohol abuse, depression, diabetes mellitus, smoking, BMI, cognitive activity, IADL, engagement in social groups, age ∗ gender, age ∗ engagement	Cognitive change. Continuous. Cognitive function measured with SIDAM Score (SISCO), Differences computed between wave2 and wave1	Functional: Social support. Dichotomous. Measured through 14-item short form of questionnaire for social support (FsozU K-14). 5-item Likert scale was adapted to include yes/no for cognitively impaired. Sum scored range 0 to 14, then dichotomised at 11.5 as cut-off . Being above the treshold indicates high perceived social support.	No association (data not shown)	n.a.

Continues in the following page

Continuing from the previous page

Study characteristics				Population characteristics			Adjustment for covariates	Outcome	Social relationship assessment	Results	
Author	Country	Study duration (yrs)	*N* in the analysis	In- and exclusion criteria	Age mean (SD), range (yrs)	Women (%)				Original from paper	OR (95% CI) in meta-analysis
Evans et al (2019)	UK	2	1498	Inclusion: Residents in two selected locations in Wales; 65+ years old. Exclusion: MMSE ≤ 25 or AGECAT dementia at baseline; AGECAT depression at baseline; living in institutions; missing data	73.2 (6.1)	50.1	Age, gender, education, social isolation, loneliness, social activity, marital status, ADL	Global cognitive function. Continuous, assessed with CAMCOG scale. Score ranges 0-107, lower scores indicate poor cognitive function	Functional: Loneliness. Dichotomous, (0 = living alone, 1 = not living alone)	Unstandardized Beta (95% CI, *p*-value): 0.03 (-0.13, 0.18, p = 0.735)	1.01 (0.94, 1.09)
Griffin et al (2020)	USA	6	7212	Inclusion: Participated in the Health and Retirement Study (HRS); 65+ years old. Exclusion: Depression at baseline	72.5 at last wave, 65+	59 at last wave	Age, sex, education, socioeconomic status, race, functional limitations, health status, cynical hostility, objective social isolation (mutual adjustment)	Cognitive function. Continuous, assessed with modified version of TICS - Telephone Interview for Cognitive Status. Range 0-35. Higher scores for higher cognitive performance	Functional: Loneliness. Continuous, assessed with the Hughes Scale based on three items. Value set to missing if more than one item was missing	Standardized Beta (95% CI), *p*-value: -0.34 (-0.56,-0.11), p <0.01	1.13 (1.04, 1.22)

Continues in the following page

Continuing from the previous page

Study characteristics				Population characteristics			Adjustment for covariates	Outcome	Social relationship assessment	Results	
Author	Country	Study duration (yrs)	*N* in the analysis	In- and exclusion criteria	Age mean (SD), range (yrs)	Women (%)				Original from paper	OR (95% CI) in meta-analysis
Hajek et al (2020)	Germany	3	3210	Inclusion: Participated in wave 5 and 6 of the German Ageing Survey (DEAS); aged 40+	65 (10.7)	50.3	Age, marital status, employment status, household net equivalent income, self-rated health, physical functioning, total number of physical illnesses. Cognitive function. Continuous, assessed with Digit Symbol test. Higher scores reflect better cognitive functioning	Functional: Perceived social isolation. Continuous, assessed with Bude and Lantermann's scale. Higher values corresponding to higher social isolation	Unstandardized Beta (95% CI): -1.13 (-1.85, -0.40)	0.83 (0.73, 0.93)	

Continues in the following page

Continuing from the previous page

Study characteristics				Population characteristics			Adjustment for covariates	Outcome	Social relationship assessment	Results	
Author	Country	Study duration (yrs)	*N* in the analysis	In- and exclusion criteria	Age mean (SD), range (yrs)	Women (%)				Original from paper	OR (95% CI) in meta-analysis
Kats et al (2016)	USA	20	13119	Inclusion: Participating to ARIC study (aged 45-64 in 1987-1989); living in Forsyth County, NC; Jackson, MS; suburbs of Minneapolis, MN and Washington County, MD. Exclusion: Not African American or Caucasian, African American in Minneapolis, African American in Washington County; missing global z-scores at baseline; missing education; missing ISEL-SF or LSNS	57 (5.7)	56.2	Age, sex, study centre, education, smoking, alcohol compsumption, hypertension, diabetes	Cognitive function. Continuous, measured with standardized global *z*-score of: Digit Symbol Sostitution Test (DSST- executive function, prcessing speed), Delayed Word Recall Test (DWRT- verbal learning, immediate memory) and Word Fluency	Functional: Social support. Dichotomous, “higher quartile” vs “lower quartile”. Measured using the short form of the Interpersonal Support Evaluation List (ISEL-SF) and Lubben Social Network Scale (LSNS). Each question of the ISEL-SF is scored on a 4-point rating scale (definitely true, probably true, probably false and definitely false; scored 0–3). ISEL-SF score has been categorized into quartiles	African Americans *z*-score (95% CI): -0.01 (-0.14, 0.12), $p = 0.84$	0.98 (0.77, 1.24)

Continues in the following page

Continuing from the previous page

Study characteristics				Population characteristics			Adjustment for covariates	Outcome	Social relationship assessment	Results	
Author	Country	Study duration (yrs)	*N* in the analysis	In- and exclusion criteria	Age mean (SD), range (yrs)	Women (%)				Original from paper	OR (95% CI) in meta-analysis
Kats et al (2016)									Functional: Social support. Dichotomous, "higher quartile" vs "lower quartile". Measured using the short form of the Interpersonal Support Evaluation List (ISEL-SF) and Lubben Social Network Scale (LSNS). Each question of the ISEL-SF is scored on a 4-point rating scale (definitely true, probably true, probably false and definitely false; scored 0–3). ISEL-SF score has been categorized into quartiles	Caucasian Americans *z*-score: (95% CI): 0.01 (-0.05, 0.05), p =0.95	1.02 (0.93, 1.12)

Continues in the following page

Continuing from the previous page

Study characteristics				Population characteristics			Adjustment for covariates	Outcome	Social relationship assessment	Results	
Author	Country	Study duration (yrs)	*N* in the analysis	In- and exclusion criteria	Age mean (SD), range (yrs)	Women (%)				Original from paper	OR (95% CI) in meta-analysis
Marioni et al (2015)	France	20	2854	Inclusion: Participated in the Paquid database; 65+ at baseline (1988); residing at home at baseline. Exclusion: Partial or absent data	77 (6.8)	59	Age, gender, education (low, medium, high), marital status	Cognitive decline. Categorical (non-decliners, moderate decliners, fast decliners). Cognitive ability assessed using MMSE, verbal fluency (Isaac's Set Test truncated at 15), abstract thinking (Wechsler Similarities Test), episodic memory and learning (Wechsler Paired Associate Test), processing speed (Digit Symbol Substitution Test), and immediate visual memory (Benton Visual Retention-Test)	Functional: Network satisfaction. Dichotomous, "satisfied" vs "not satisfied", assessed with 4 response Likert scale	HR (95% CI): 0.79 (0.63, 0.99)	0.95 (0.77, 1.17)

Continues in the following page

Continuing from the previous page

Study characteristics				Population characteristics			Adjustment for covariates	Outcome	Social relationship assessment	Results	
Author	Country	Study duration (yrs)	*N* in the analysis	In- and exclusion criteria	Age mean (SD), range (yrs)	Women (%)				Original from paper	OR (95% CI) in meta-analysis
McHugh Power et al (2019)	Ireland	4	7433	Inclusion: 50+ years old or spouses of participants 50+ years old. Exclusion: Diagnosis of dementia; Alzheimer's disease; organic brain syndrome; senility; serious memory impairment; any emotional, nervous, or psychiatric problem	63.9 (9.8), 50-105	53.3	Age, sex, education, physical health, depressive symptoms, anxiety symptoms, cardiovascular disease	Global cognitive functioning. Continuous, assessed with immediate and delayed recall, MMSE, verbal fluency	Functional: Loneliness. Continuous, assessed with a 5-item version of UCLA loneliness scale	Standardized Beta (SE): -0.103 (SE=0.025), p <0.001	1.45 (1.21, 1.74)
Noguchi et al (2019)	Japan	1	121	Inclusion: Living in Togo town, Japan; enrolled in municipal health-check project. Exclusion: Age <65; history of diagnosis of dementia or mental illness; non-independent walking ability; missing data for cognitive function	73.8 (4.9), 65+	47.1	Age, sex, BMI, living alone, equivalent income, medical history, depression, IADL, walking speed, walking time	Cognitive function. Continuous, assessed with MoCa-J at baseline and follow-up. Total score ranges 0 to 30, higher scores indicate higher cognitive function	Functional: Social support. Measured with Two-Way Social Support Scale. Three sources of social support were identified: coresiding family, non-coresiding family and relatives, neighbors and friends. Coresiding family	Unstandardized Beta (95% CI): 0.28 (-2.05, 2.61)	1.08 (0.38, 3.08)

Continues in the following page

Continuing from the previous page

Study characteristics				Population characteristics			Adjustment for covariates	Outcome	Social relationship assessment	Results	
Author	Country	Study duration (yrs)	*N* in the analysis	In- and exclusion criteria	Age mean (SD), range (yrs)	Women (%)				Original from paper	OR (95% CI) in meta-analysis
Noguchi et al (2019)										Non-coresiding family Unstandardized Beta (95% CI): 0.51 (-0.33, 1.35)	1.35 (0.82, 2.21)
Noguchi et al (2019)										Neighbors and friends Unstandardized Beta (95% CI): 1.23 (0.36, 2.09)	2.07 (1.22, 3.52)

Continues in the following page

Continuing from the previous page

Study characteristics				Population characteristics			Adjustment for covariates	Outcome	Social relationship assessment	Results	
Author	Country	Study duration (yrs)	*N* in the analysis	In- and exclusion criteria	Age mean (SD), range (yrs)	Women (%)				Original from paper	OR (95% CI) in meta-analysis
Pillemer et al (2019)	USA	5	30	Inclusion: Residing in Westchester County, New York, USA; 65+ years old; English-speaking Exclusion: Severe auditory or visual disturbances interfering with testing; mobility limitations; medical/neurological history interfering with performance; diagnosis of dementia	76.5 (6.6), 65-95	57.2	Age, education, gender, ethnicity, depressive symptoms (GDS), disease comorbidity	Cognitive decline. Continuous, assessed with the RBANS battery	Functional: Social support. Continuous, assessed with MOS-SSS scale of 19 items measuring social support	HR (95% CI, *p*-value) 2.06 (1.16, 3.65, p = 0.013)	0.49 (0.27, 0.86)

Continues in the following page

Continuing from the previous page

Study characteristics				Population characteristics			Adjustment for covariates	Outcome	Social relationship assessment	Results	
Author	Country	Study duration (yrs)	*N* in the analysis	In- and exclusion criteria	Age mean (SD), range (yrs)	Women (%)				Original from paper	OR (95% CI) in meta-analysis
Rawtaer et al (2017)	Singapore	8	1601	Inclusion: Singaporean residents in the south-east region of Singapore; aged 55+. Exclusion: MCI or dementia at baseline; death or loss to follow-up	64.9 (6.8), 55+	64.5	Age, sex, ethnicity, education, smoking, alcohol consumption, depression, APOE4 status, physical activity, social activities tertile score (SAS), productive activities tertile score (PAS), leisure-time activity score, living alone, marital status, satisfaction with life	Cognitive decline. Dichotomized, measured with MMSE. Cognitive decline if MMSE<50% percentile value estimated for the individual given age, education, prior MMSE score and interval years between measurements	Functional: Loneliness. Dichotomous, measured with 3-category scale ([1] not at all lonely; [2] faily lonely; [3] very lonely) and reshaped in yes vs no in feeling lonely	OR (95% CI): 1.06 (0.76, 1.4), *p*-value=0.74	1.06 (0.76, 1.46)
Tomioka et al (2018)	Japan	3	6093	Inclusion: 65+ years old; living in the Nara Prefecture, residing in community-dwelling, not certified as “dependent in daily living activities”	72.8, 65-96	54.6	Age, family structure, BMI, pensions, number of medications used, medical condition, drinking, smoking, depression, IADL, ADL	Cognitive decline. Dichotomized, cognitive function assessed using the Cognitive Performance Scale	Functional: Social support. Measured through participation in five types of group involvement: neighborhood associations, hobby groups, local event groups, senior citizens clubs, volunteer groups	Neighbourhood Men OR (95% CI): 0.81 (0.66,0.99) Women OR (95% CI): 0.93 (0.74,1.17)	Men: 1.23 (1.01, 1.51) Women: 1.08 (0.86, 1.35)

Continues in the following page

Continuing from the previous page

Study characteristics				Population characteristics			Adjustment for covariates	Outcome	Social relationship assessment	Results	
Author	Country	Study duration (yrs)	*N* in the analysis	In- and exclusion criteria	Age mean (SD), range (yrs)	Women (%)				Original from paper	OR (95% CI) in meta-analysis
Tomioka et al (2018)										Hobby groups Men OR (95% CI): 0.96 (0.74, 1.25) Women OR (95% CI): 0.70 (0.54, 0.91)	Men: 1.04 (0.80, 1.35) Women: 1.43 (1.10, 1.85)
Tomioka et al (2018)										Senior citizens clubs Men OR (95% CI): 1.16 (0.91, 1.49) Women OR (95% CI): 0.97 (0.78, 1.22)	Men: 1.27 (1.01, 1.59) Women: 1.14 (0.89, 1.45)
Tomioka et al (2018)										Volunteer groups Men OR (95% CI): 0.95 (0.71, 1.27) Women OR (95% CI): 0.66 (0.45, 0.96)	Men: 0.86 (0.67, 1.10) Women: 1.03 (0.82, 1.29)

Continues in the following page

Continuing from the previous page

Study characteristics				Population characteristics			Adjustment for covariates	Outcome	Social relationship assessment	Results	
Author	Country	Study duration (yrs)	*N* in the analysis	In- and exclusion criteria	Age mean (SD), range (yrs)	Women (%)				Original from paper	OR (95% CI) in meta-analysis
Tomioka et al (2018)										Local event groups Men OR (95% CI): 0.79 (0.63, 0.99) Women OR (95% CI): 0.88 (0.69, 1.12)	Men: 1.05 (0.79, 1.41) Women: 1.52 (1.04, 2.21)
Wang et al (2019)	UK	20	657	Inclusion: Wave 3-7 of the CC75C Study. Exclusion: Participation to concurrent study	86 (4)	71	Age, sex, education	Cognitive function. Categorical, assessed with MMSE (range 0-30). Four categories MMSE: normal cognition (26-30), mild cognitive impairment (22-25), moderate impairment (18-21), sever impairment (0-17)	Functional: Loneliness. Categorical, assessed with item “Do you feel lonely?”.	Unstandardized Beta (95% CI): -0.6 (-1.5, 0.4)	n.a.

Continues in the following page

Continuing from the previous page

Study characteristics				Population characteristics			Adjustment for covariates	Outcome	Social relationship assessment	Results	
Author	Country	Study duration (yrs)	*N* in the analysis	In- and exclusion criteria	Age mean (SD), range (yrs)	Women (%)				Original from paper	OR (95% CI) in meta-analysis
Zhong et al (2017)	China	9	2456	Inclusion: Aged 65-105; being sampled in the Chinese Longitudinal Health Longevity Survey. Exclusion: Death during follow-up; missing data	83.8 (7.7), 65-105	52.6	Age, gender, education (0 = no schooling; 1 = some schooling (equal or more than 1 year)), phisical exercise, smoking, participation in social activities, marital status, living arrangement	Cognitive function. Continuous, assessed with Chines MMSE (reduced form obtained deleting 2 items of language, 1 item of time orientation, 4 items of orientation to place). Higher scores for better cognition	Functional: Loneliness. Continuous, assessed with single-item self-reported measure based on the modified de Jong-Gierveld scale. Range: 1-5. Higher scores for more loneliness	Unstandardized Beta: -0.153, *p*-value<.001	0.91 (n.a.)
Zhou et al (2019)	China	4	6898	Inclusion: Participated in the Chinese Longitudinal Healthy Longevity Study between 2008-2011. Exclusion: Age <65 at baseline; cognitive impairment at baseline	Range: 65-95	50.8	Age, education level, employment status, BMI, ADL disability, cardiovascular disease, diabetes, physical activity, drinking, smoking, marital status, living alone, social support	Cognitive impairment. Dichotomized, measured using the Chinese revised version of MMSE (0-30). Cutoff point of ¡18	Functional: Loneliness. Dichotomous, measured with 5-category scale for question "Do you feel lonely?" ([1] never; [2] rarely; [3] sometimes; [4] often; [4] always) and reshaped in "lonely" ([3]+[4]+[5]) vs "not lonely" ([1]+[2])	OR (95% CI): 1.30 (1.01, 1.69)	1.30 (1.01, 1.69)

Concluding from the previous page

Table 3: Combination of aspects of social relationship.

Study characteristics				Population characteristics			Adjustment for covariates	Outcome	Social relationship assessment	Results	
Author	Country	Study duration (yrs)	*N* in the analysis	In- and exclusion criteria	Age mean (SD), range (yrs)	Women (%)				Original from paper	OR (95% CI) in meta-analysis
Armstrong et al (2015)	USA	6	2759	Inclusion: Participated in the Honolulu-Asia Ageing Study (HAAS); being Japanese-American men; born 1900-1919; lived in Oahu at baseline. Exclusion: Dementia at baseline	77.9 (4.7), 71-93	0	Age, education (years), baseline cognitive state	Cognitive function. Categorical, assessed with the CASI scale (100-point scale). Individual scores reversed, lower scores indicate higher cognition levels. Finally, scores have been grouped by intervals of three (i.e. 0-2, 3-5, etc.)	Composite: Social vulnerability. Continuous, composite index of 18 social variables from baseline wave. Score ranges 0 to 1	Standardized Beta (95% CI): 0.14 (0.05,0.21)	
Evans et al (2018)	Wales	2	2224	Inclusion: Living in Gwynedd and Ynys Mon or Neath Port Talbot in Wales; aged 65+. Exclusion: Cognitive impairment at baseline; dementia at baseline; depression at baseline; living in an institution; missing data on baseline or follow-up	72.7 (6.2), 65-100	50.6	Age, gender, education (years), sensory problems, require help with daily tasks. Cognitive function. Continuous, assessed with CAMCOG test at baseline and follow-up	Range (0-107)	Combination: Lubben Social Network Scale. Continuous, range (0-30). Higher scores indicat lower social isolation	Unstandardized Beta (95% CI), *p*-value: 0.05(0.01,0.10), *p* <0.03	1.09 (1.02, 1.18)

Continues in the following page

Continuing from the previous page

Study characteristics				Population characteristics			Adjustment for covariates	Outcome	Social relationship assessment	Results	
Author	Country	Study duration (yrs)	*N* in the analysis	In- and exclusion criteria	Age mean (SD), range (yrs)	Women (%)				Original from paper	OR (95% CI) in meta-analysis
Marioni et al (2015)	France	20	2854	Inclusion: Participated in the Paquid database; 65+ at baseline (1988); residing at home at baseline. Exclusion: Partial or absent data	77 (6.8)	59	Age, gender, education (low, medium, high), marital status	Cognitive decline. Categorical (non-decliners, moderate decliners, fast decliners). Cognitive ability assessed using MMSE, verbal fluency (Isaac's Set Test truncated at 15), abstract thinking (Wechsler Similarities Test), episodic memory and learning (Wechsler Paired Associate Test), processing speed (Digit Symbol Substitution Test), and immediate visual memory (Benton Visual Retention-Test)	Combination: Social engagement. Categorized upon tertiles. Assessed with twelve questions in four domains: social, intellectual and physical engagement; network size; satisfaction with social relationship; self-perception of feeling well understood	HR (95% CI): 0.79 (0.63, 0.99), $p < 0.05$	1.27 (1.01, 1.59)

Continues in the following page

Continuing from the previous page

Study characteristics				Population characteristics			Adjustment for covariates	Outcome	Social relationship assessment	Results	
Author	Country	Study duration (yrs)	*N* in the analysis	In- and exclusion criteria	Age mean (SD), range (yrs)	Women (%)				Original from paper	OR (95% CI) in meta-analysis
Murayama et al (2013)	Japan	2	681	Inclusion: Residing in community-dwelling in Hatoyama; 65+ years old. Exclusion: Long-term care certification (levels 1-5) admitted to hospitals or residing in nursing homes; not participating to follow-up	71.8 (5.1)	42.1	Age, gender, marital status, SES (education, long-term occupation), lifestyle factors (smoking, body.mass index), comorbidity (hypertension, cardiovascular diseases, hyperlipidemia, cerebrovascular diseases, diabetes mellitus), functional capacity	Cognitive decline. Continuous (range 0-30), measured with MMSE at baseline and at follow-up	Combination: Bonding social capital. Dichotomous (agree vs disagree). Assessed as perceived homogeneity of personal network by asking participants if they agree that they have some networks with people of similar social characteristics to them on daily basis	OR (95% CI): 0.96 (0.5, 1.9)	0.96 (0.5, 1.9)

Continues in the following page

Continuing from the previous page

Study characteristics				Population characteristics			Adjustment for covariates	Outcome	Social relationship assessment	Results	
Author	Country	Study duration (yrs)	*N* in the analysis	In- and exclusion criteria	Age mean (SD), range (yrs)	Women (%)				Original from paper	OR (95% CI) in meta-analysis
Murayama et al (2013)										Combination: Bridging social capital. Dichotomous (agree vs disagree). Assessed as perceived heterogeneity of personal network by asking participants if they agree that they have some networks with people of dissimilar social characteristics to them on daily basis	OR (95% CI): 1.11 (0.5,2.2)
0.90 (0.43, 1.89)											

Continues in the following page

Continuing from the previous page

Study characteristics				Population characteristics			Adjustment for covariates	Outcome	Social relationship assessment	Results	
Author	Country	Study duration (yrs)	*N* in the analysis	In- and exclusion criteria	Age mean (SD), range (yrs)	Women (%)				Original from paper	OR (95% CI) in meta-analysis
Zhou et al (2019)	China	4	6998	Inclusion: Participated in the Chinese Longitudinal Healthy Longevity Study between 2008-2011. Exclusion: Age <65 at baseline; cognitive impairment at baseline	80.9 (10.1)	51.2	Age, gender, education (years), BMI, ADL disability, cardiovascular disease, physical activity, drinking, smoking, working status	Cognitive impairment. Dichotomized, measured using the Chinese revised version of MMSE (0-30). Cutoff point of <18	Combination: Social engagement. Continuous, measured with five dichotomous items (marital status, living arrangement, avalaibility of help, availability of confident, participation in social activities). Higher scores indicate better social engagement	OR (95% CI): 0.89 (0.82, 0.97)	0.89 (0.82, 0.97)

Concluding from the previous page

3. Funnel plots

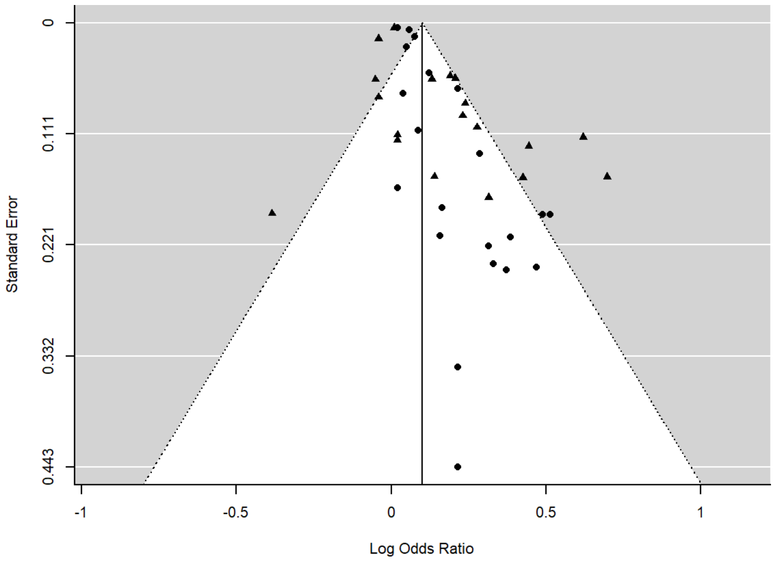

Figure 1: Funnel plots of structural aspects of social relationships as predictor of cognitive decline.

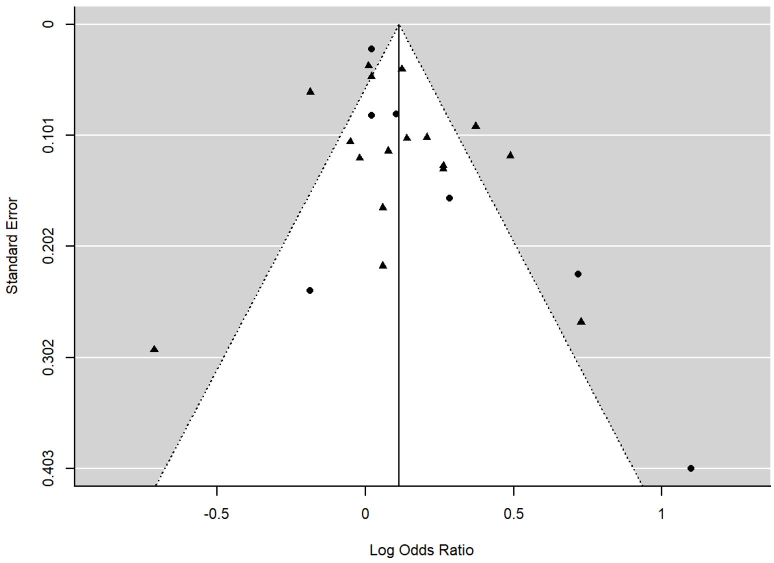

Figure 2: Funnel plots of functional aspects of social relationships as predictor of cognitive decline.

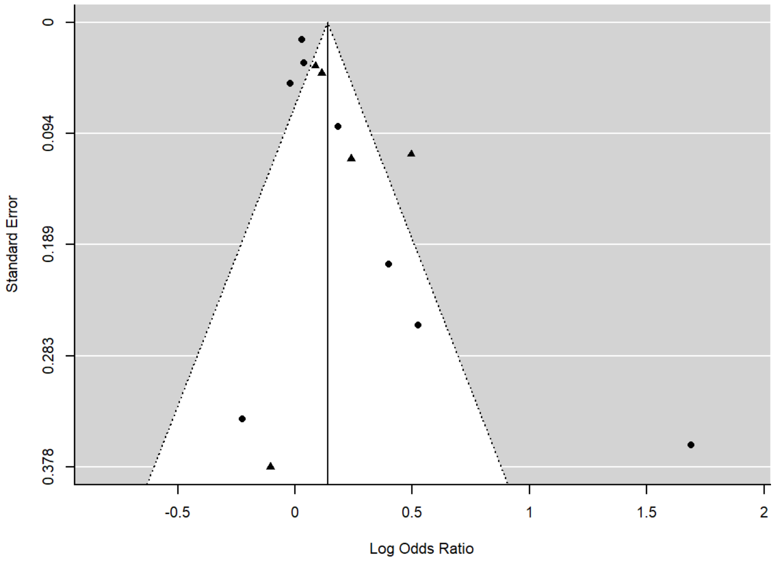

Figure 3: Funnel plots of combination of structural and functional aspects of social relationships as predictor of cognitive decline.

4. Subgroup analyses

Table 4: Stratified analyses of structural aspects of social relationships.

Strata	Number of studies	OR (95% CI)	I^2, p-value for hetero-geneity	p-value for subgroup differences
**Publication year**				.69
Before 2006	10	1.08 (1.03-1.13)	31%, .16	
2007–2011	11	1.11 (1.05-1.18)	75%, <.01	
2012–2018	14	1.14 (1.07-1.22)	86%, <.01	
After 2019	5	1.18 (1.04-1.34)	73%, .01	
**Being included in Kuiper et al (2016)**				.64
Yes	21	1.09 (1.05-1.12)	70%, <.01	
No	19	1.16 (1.09-1.24)	87%, <.01	
**Study geographic area**				.13
Asia	15	1.16 (1.08-1.26)	60%, <.01	
Europe	11	1.28 (1.10-1.48)	88%, <.01	
America	14	1.06 (1.03-1.09)	85%, <.01	
Study size (participants)				<.01
≤687	9	1.43 (1.24-1.66)	0%, .98	
>687 and ≤1635	10	1.13 (1.07-1.20)	80%, <.01	
>1635 and ≤3413	8	1.16 (1.07-1.26)	45%, .08	
>3413	13	1.07 (1.02-1.12)	90%, <.01	
Study follow-up duration (years)				.03
≤3	10	1.13 (1.06-1.21)	73%, <.01	
>3 and ≤5	8	1.41 (1.13-1.75)	67%, <.01	
>5 and ≤9	8	1.13 (1.06-1.21)	80%, <.01	
>9	14	1.09 (1.03-1.15)	80%, <.01	
**Age of study participants (years)**				.94
≤65	10	1.21 (1.06-1.37)	88%, <.01	
66–74	16	1.12 (1.07-1.17)	82%, <.01	
≥75	14	1.12 (1.06-1.18)	78%, <.01	
**Outcome**				.19
Cognitive function	10	1.25 (1.11-1.40)	89%, <.01	
Cognitive decline	30	1.09 (1.06-1.13)	77%, <.01	
**Type of outcome**				.53
Continuous	23	1.10 (1.06-1.13)	87%, <.01	
Dichotomous	17	1.15 (1.06-1.24)	58%, <.01	
Social relationship measurement				<.01
Low social activity	28	1.17 (1.12-1.21)	82%, <.01	
Small social network size	12	1.05 (1.00-1.10)	75%, <.01	

Table 5: Stratified analyses of functional aspects of social relationships.

Strata	Number of studies	OR (95% CI)	I^2, p-value for heterogeneity	p-value for subgroup differences
**Publication year**				.81
Before 2006	4	1.26 (0.98-1.64)	55%, .08	
2007–2011	4	1.09 (0.90-1.31)	70%, .02	
2012–2018	9	1.13 (1.02-1.26)	59%, .01	
After 2019	7	1.10 (0.93-1.29)	87%, <.01	
**Being included in Kuiper et al (2016)**				.74
Yes	8	1.15 (1.00-1.32)	66%, <.01	
No	16	1.12 (1.02-1.22)	78%, <.01	
**Study geographic area**				.40
Asia	8	1.20 (1.10-1.32)	0%, .45	
Europe	6	1.19 (0.96-1.48)	88%, <.01	
America	10	1.08 (0.99-1.17)	70%, <.01	
Study size (participants)				.54
≤687	5	1.18 (0.68-2.04)	81%, <.01	
>687 and ≤1635	6	1.03 (0.99-1.06)	0%, .56	
>1635 and ≤3413	5	1.13 (0.89-1.44)	84%, <.01	
>3413	8	1.19 (1.07-1.33)	72%, <.01	
**Study follow-up duration (years)**				.96
≤3	8	1.13 (0.97-1.31)	78%, <.01	
>3 and ≤5	5	1.05 (0.83-1.34)	83%, <.01	
>5 and ≤9	3	1.14 (1.05-1.23)	0%, .55	
>9	8	1.15 (1.00-1.31)	72%, <.01	
**Age of study participants (years)**				.21
≤65	6	1.04 (0.89-1.21)	80%, <.01	
66–74	11	1.20 (1.09-1.33)	70%, <.01	
≥75	7	1.09 (0.91-1.31)	70%, <.01	
**Outcome**				.57
Cognitive function	9	1.10 (0.97-1.25)	86%, <.01	
Cognitive decline	15	1.13 (1.04-1.23)	54%, <.01	
**Type of outcome**				.16
Continuous	14	1.08 (0.99-1.17)	79%, <.01	
Dichotomous	10	1.19 (1.07-1.34)	51%, .03	
**Social relationship measurement**				.45
Loneliness	10	1.18 (1.05-1.32)	86%, <.01	
Low social/emotional support	12	1.10 (1.00-1.21)	51%, .02	
other	2	0.97 (0.80-1.17)	0%, .65	

Table 6: Stratified analyses of combinations of functional and structural aspects of social relationships.

Strata	Number of studies	OR (95% CI)	I^2, p-value for heterogeneity	p-value for subgroup differences
**Publication year**				<.01
Before 2006	3	2.26 (1.15-4.47)	80%, .01	
2007–2011	5	1.03 (1.00-1.07)	12%, .34	
2012–2018	4	1.26 (1.00-1.58)	77%, <.01	
After 2019	1	1.12 (1.03-1.22)	–	
**Being included in Kuiper et al (2016)**				.38
Yes	8	1.12 (1.01-1.24)	79%, <.01	
No	5	1.20 (1.06-1.36)	70%, .01	
**Study geographic area**				<.01
Asia	2	1.12 (1.03-1.21)	0%, .57	
Europe	4	1.07 (1.01-1.14)	22%, .28	
America	5	1.17 (1.02-1.35)	83%, <.01	
**Study size (participants)**				<.01
≤687	4	1.85 (1.01-3.38)	78%, <.01	
>687 and ≤1635	2	0.98 (0.88-1.08)	0%, .55	
>1635 and ≤3413	5	1.12 (1.03-1.22)	81%, <.01	
>3413	2	1.13 (1.05-1.22)	0%, .48	
**Study follow-up duration (years)**				.03
≤3	4	1.67 (0.89-3.14)	87%, <.01	
>3 and ≤5	3	1.06 (0.98-1.14)	50%, .14	
>5 and ≤9	2	1.60 (1.32-1.95)	0%, .68	
>9	4	1.07 (0.98-1.18)	56%, .08	
**Age of study participants (years)**				.27
≤65	1	0.98 (0.89-1.08)	–	
66–74	3	1.10 (1.03-1.18)	0%, .52	
≥75	9	1.23 (1.09-1.38)	84%, <.01	
**Outcome**				.30
Cognitive function	2	1.32 (0.88-1.97)	92%, <.01	
Cognitive decline	11	1.12 (1.03-1.22)	74%, <.01	
Type of outcome				.03
Continuous	4	1.05 (1.00-1.09)	12%, .39	
Dichotomous	9	1.30 (1.13-1.51)	85%, <.01	
Social relationship measurement				.04
Social support	8	1.34 (1.14-1.58)	86%, <.01	
Social network	5	1.05 (1.00-1.58)	31%, .22	

5. Code for the analysis

This is the *R* code used to perform the published analysis.

```
library(tidyverse)
library(metafor)

#### STRUCTURAL ASPECTS ####
db_structural <- db %>% filter(type_social_rel=="structural")
db_structural_ma <- db_structural %>%
                    slice(-c(2,11,13,14,16,17)) %>%
                    filter(!is.na(OR) & !is.na(OR_INF) & !is.na(OR_SUP))

#selection of studies reporting multiple social relationships proxy variables
```

```
db_structural_ma <- db_structural_ma %>% arrange(year_pub,reference)

# Meta-analysis
ma_structural <- rma.uni(yi=log(OR),
                sei=(log(OR_SUP)-log(OR_INF))/3.92,
                measure="OR",slab=reference,data=db_structural_ma,method="DL")

# Forest-plot
forest(ma_structural,
at=log(c(.25,.5,1,2,4)),xlim=c(-6.0,2.5),refline=0,
atransf=exp,
header="Author␣(Year)",
ilab=cbind(db_structural_ma$country,db_structural_ma$n,db_structural_ma$gender,
db_structural_ma$social_relationship),
ilab.xpos=c(-4.5,-3.5,-3.0,-2.5),ilab.pos=4,cex=.7,
mlab="Pooled␣Random-effects␣OR␣estimate")

# Headings
text(-4.5,42,"Country",pos=4,font=2,cex=.7)
text(-3.5,43,"Study",pos=4,font=2,cex=.7)
text(-3.5,42,"size",pos=4,font=2,cex=.7)
text(-3,42,"Gender",pos=4,font=2,cex=.7)
text(-2.5,42,"Social␣relationship␣assessment",pos=4,font=2,cex=.7)

# Heterogeneity
text(-6.0,-2,bquote("Heterogeneity:"~I^2~"="~.(round(ma_structural$I2,0))~"%"~p~.
(ifelse(ma_structural$QEp<.01,"<.01",.(round(ma_structural$QEp,2))))),pos=4,font=1,
cex=.7)

# Cumulative meta-analysis
ma_cumulative_structural <- cumul(ma_structural)

# Cumulative meta-analysis forest-plot
forest.cumul.rma(ma_cumulative_structural,
at=log(c(.25,.5,1,2,4)),xlim=c(-5.5,2),refline=0,
atransf=exp,
header="Author␣(Year)",
ilab=cbind(db_structural_ma$country,db_structural_ma$n,db_structural_ma$gender,
db_structural_ma$social_relationship),
ilab.xpos=c(-4,-3,-2.5,-2.0),ilab.pos=4,cex=.7)

# Headings
text(-4,42,"Country",pos=4,font=2,cex=.7)
text(-3,43,"Study",pos=4,font=2,cex=.7)
text(-3,42,"size",pos=4,font=2,cex=.7)
text(-2.5,42,"Gender",pos=4,font=2,cex=.7)
text(-2,42,"Social␣relationship␣assessment",pos=4,font=2,cex=.7)

# Funnel plot
funnel(ma_structural,pch=c(rep(17,18),rep(16,21),17))
```

```
#Egger test
regtest(ma_structural,model="rma")

#### FUNCTIONAL ASPECTS ####
db_functional  <- db %>% filter(type_social_rel=="functional")

#selection of studies reporting multiple social relationships
db_functional_ma <- db_functional %>%
                    slice(-c(12,13,14,15,16,17,18,19,22,23))
                    %>% filter(!is.na(OR) & !is.na(OR_INF) & !is.na(OR_SUP))

db_functional_ma <- db_functional_ma %>% arrange(year_pub,reference)

# Meta-analysis
ma_functional <- rma.uni(yi=log(OR),
                sei=(log(OR_SUP)-log(OR_INF))/3.92,
                measure="OR",slab=reference,data=db_functional_ma,method="DL")

# Forest plot
forest(ma_functional,
at=log(c(.25,.5,1,2,4)),xlim=c(-6.0,2.5),refline=0,
atransf=exp,
header="Author␣(Year)",
ilab=cbind(db_functional_ma$country,db_functional_ma$n,db_functional_ma$gender,
db_functional_ma$social_relationship),
ilab.xpos=c(-4.5,-3.5,-3.0,-2.5),ilab.pos=4,cex=.7,
mlab="Pooled␣Random-effects␣OR␣estimate")

# Headings
text(-4.5,26,"Country",pos=4,font=2,cex=.7)
text(-3.5,27,"Study",pos=4,font=2,cex=.7)
text(-3.5,26,"size",pos=4,font=2,cex=.7)
text(-3,26,"Gender",pos=4,font=2,cex=.7)
text(-2.5,26,"Social␣relationship␣assessment",pos=4,font=2,cex=.7)

# Heterogeneity
text(-6.0,-2,bquote("Heterogeneity:"~I^2~"="~.(round(ma_functional$I2,0))~"%"~p~.
(ifelse(ma_functional$QEp<.01,"<.01",.(round(ma_functional$QEp,2))))),pos=4,font=1,
cex=.7)

# Cumulative meta-analysis
ma_cumulative_functional <- cumul(ma_functional)

# Cumulative meta-analysis forest-plot
forest.cumul.rma(ma_cumulative_functional,
at=log(c(.25,.5,1,2,4)),xlim=c(-5.5,2),refline=0,
atransf=exp,
header="Author␣(Year)",
ilab=cbind(db_functional_ma$country,db_functional_ma$n,db_functional_ma$gender,
db_functional_ma$social_relationship),
ilab.xpos=c(-4,-3,-2.5,-2.0),ilab.pos=4,cex=.7)
```

```r
# Headings
text(-4,26,"Country",pos=4,font=2,cex=.7)
text(-3,27,"Study",pos=4,font=2,cex=.7)
text(-3,26,"size",pos=4,font=2,cex=.7)
text(-2.5,26,"Gender",pos=4,font=2,cex=.7)
text(-2,26,"Social␣relationship␣assessment",pos=4,font=2,cex=.7)

# Funnel plot
funnel(ma_functional,pch=c(rep(17,16),rep(16,7),17))

#Egger test
regtest(ma_functional,model="rma")

#### COMBINATION (FUNCTIONAL AND STRUCTURAL) ASPECTS ####
db_combination <- db %>% filter(type_social_rel=="combination")

#selection of studies reporting multiple social relationships proxy variables
db_combination_ma <- db_combination %>%
                     slice(-2) %>%
                     filter(!is.na(OR) & !is.na(OR_INF) & !is.na(OR_SUP))

db_combination_ma <- db_combination_ma %>% arrange(year_pub,reference)

# Meta-analysis
ma_combination <- rma.uni(yi=log(OR),
                sei=(log(OR_SUP)-log(OR_INF))/3.92,
                measure="OR",slab=reference,data=db_combination_ma,method="DL")

# Forest plot
forest(ma_combination,
at=log(c(.25,.5,1,2,4)),xlim=c(-6.0,2.5),refline=0,
atransf=exp,
header="Author␣(Year)",
ilab=cbind(db_combination_ma$country,db_combination_ma$n,db_combination_ma$gender,
db_combination_ma$social_relationship),
ilab.xpos=c(-4.5,-3.5,-3.0,-2.5),ilab.pos=4,cex=.7,
mlab="Pooled␣Random-effects␣OR␣estimate")

# Headings
text(-4.5,15,"Country",pos=4,font=2,cex=.7)
text(-3.5,16,"Study",pos=4,font=2,cex=.7)
text(-3.5,15,"size",pos=4,font=2,cex=.7)
text(-3,15,"Gender",pos=4,font=2,cex=.7)
text(-2.5,15,"Social␣relationship␣assessment",pos=4,font=2,cex=.7)

# Heterogeneity
text(-6.0,-2,bquote("Heterogeneity:"~I^2~"="~.(round(ma_combination$I2,0))~"%"~p~.
(ifelse(ma_combination$QEp<.01,"<.01",.(round(ma_combination$QEp,2))))),pos=4,font=1,
cex=.7)
```

```r
# Cumulative meta-analysis
ma_cumulative_combination <- cumul(ma_combination)

# Cumulative meta-analysis forest-plot
forest.cumul.rma(ma_cumulative_combination,
at=log(c(.25,.5,1,2,4)),xlim=c(-5.5,2.5),refline=0,
atransf=exp,
header="Author␣(Year)",
ilab=cbind(db_combination_ma$country,db_combination_ma$n,
db_combination_ma$gender,
db_combination_ma$social_relationship),
ilab.xpos=c(-4,-3,-2.5,-2.0),ilab.pos=4,cex=.7)

# Headings
text(-4,15,"Country",pos=4,font=2,cex=.7)
text(-3,16,"Study",pos=4,font=2,cex=.7)
text(-3,15,"size",pos=4,font=2,cex=.7)
text(-2.5,15,"Gender",pos=4,font=2,cex=.7)
text(-2,15,"Social␣relationship␣assessment",pos=4,font=2,cex=.7)

# Funnel plot
funnel(ma_combination,pch=c(rep(17,5),rep(16,8)))

# Egger test
regtest(ma_combination,model="rma")
```

References

Ali T, Nilsson C, Weuve J, Rajan K, de Leon C (2018) Effects of social network diversity on mortality, cognition and physical function in the elderly: a longitudinal analysis of the chicago health and aging project (chap). Journal of Epidemiology & Community Health 72(11):990–996, URL http://dx.doi.org/10.1136/jech-2017-210236

Armstrong J, Mitnitski A, Andrew M, Launer L, White L, Rockwood K (2015) Cumulative impact of health deficits, social vulnerabilities, and protective factors on cognitive dynamics in late life: a multistate modeling approach. Alzheimer's Research & Therapy 7(1):1–9, DOI https://doi.org/10.1186/s13195-015-0120-7

Bourassa K, Memel M, Woolverton C, Sbarra D (2017) Social participation predicts cognitive functioning in aging adults over time: comparisons with physical health, depression, and physical activity. Aging & mental health 21(2):133–146, DOI https://doi.org/10.1080/13607863.2015.1081152

Chen T, Chang H (2016) Developmental patterns of cognitive function and associated factors among the elderly in taiwan. Scientific reports 6(1):1–10, DOI https://doi.org/10.1038/srep33486

Donovan N, Wu Q, Rentz D, Sperling R, Marshall G, Glymour M (2017) Loneliness, depression and cognitive function in older us adults. International journal of geriatric psychiatry 32(5):564–573, DOI https://doi.org/10.1002/gps.4495

Eisele M, Zimmermann T, Köhler M, Wiese B, Heser K, Tebarth F, Weeg D, Olbrich J, Pentzek M, Fuchs A, et al (2012) Influence of social support on cognitive change and mortality in old age: results from the prospective multicentre cohort study agecode. BMC geriatrics 12(1):1–13, DOI https://bmcgeriatr.biomedcentral.com/articles/10.1186/1471-2318-12-9

Ellwardt L, Van Tilburg T, Aartsen M (2015) The mix matters: Complex personal networks relate to higher cognitive functioning in old age. Social science & medicine 125:107–115, DOI https://doi.org/10.1016/j.socscimed.2014.05.007

Elovainio M, Sommerlad A, Hakulinen C, Pulkki-Råback L, Virtanen M, Kivimäki M, Singh-Manoux A (2017) Structural social relations and cognitive ageing trajectories: Evidence from the whitehall ii cohort study. International Journal of Epidemiology 47(3):701–708, DOI https://doi.org/10.1093/ije/dyx209

Evans I, Llewellyn D, Matthews F, Woods R, Brayne C, Clare L, Team CWR (2018) Social isolation, cognitive reserve, and cognition in healthy older people. PloS one 13(8):e0201,008, DOI https://doi.org/10.1371/journal.pone.0201008

Evans I, Llewellyn D, Matthews F, Woods R, Brayne C, Clare L, Team CWR, et al (2019) Living alone and cognitive function in later life. Archives of gerontology and geriatrics 81:222–233, DOI https://doi.org/10.1016/j.archger.2018.12.014

Griffin S, Mezuk B, Baylor Williams A, Perrin P, Rybarczyk B (2020) Isolation, not loneliness or cynical hostility, predicts cognitive decline in older americans. Journal of Aging and Health 32(1-2):52–60, DOI https://doi.org/10.1177/0898264318800587

Hajek A, Riedel-Heller S, König H (2020) Perceived social isolation and cognitive functioning. longitudinal findings based on the german ageing survey. International journal of geriatric psychiatry 35(3):276–281, DOI https://doi.org/10.1002/gps.5243

Haslam C, Cruwys T, Haslam S (2014) "the we's have it": Evidence for the distinctive benefits of group engagement in enhancing cognitive health in aging. Social Science & Medicine 120(nil):57–66, DOI https://doi.org/10.1016/j.socscimed.2014.08.037

Hwang J, Park S, Kim S (2018) Effects of participation in social activities on cognitive function among middle-aged and older adults in korea. International journal of environmental research and public health 15(10):2315, DOI doi:10.3390/ijerph15102315

Kats D, Patel M, Palta P, Meyer M, Gross A, Whitsel E, Knopman D, Alonso A, Mosley T, Heiss G (2016) Social support and cognition in a community-based cohort: the atherosclerosis risk in communities (aric) study. Age and ageing 45(4):475–480, DOI https://doi.org/10.1093/ageing/afw060

Kuiper J, Zuidersma M, Zuidema S, Burgerhof J, Stolk R, Oude Voshaar R, Smidt N (2016) Social relationships and cognitive decline: a systematic review and meta-analysis of longitudinal cohort studies. International Journal of Epidemiology 45(4):1169–1206, DOI https://doi.org/10.1093/ije/dyw089

Lee H, Ang S (2020) Productive activities and risk of cognitive impairment and depression: Does the association vary by gender? Sociological Perspectives 63(4):608–629, DOI https://doi.org/10.1177/0731121419892622

Marioni R, Proust-Lima C, Amieva H, Brayne C, Matthews F, Dartigues J, Jacqmin-Gadda H (2015) Social activity, cognitive decline and dementia risk: a 20-year prospective cohort study. BMC public health 15(1):1–8, DOI https://doi.org/10.1186/s12889-015-2426-6

McHugh Power J, Tang J, Kenny R, Lawlor B, Kee F (2019) Mediating the relationship between loneliness and cognitive function: the role of depressive and anxiety symptoms. Aging & Mental Health 24(7):1071–1078, DOI https://doi.org/10.1080/13607863.2019.1599816

Min J (2018) A longitudinal study of cognitive trajectories and its factors for koreans aged 60 and over: A latent growth mixture model. International journal of geriatric psychiatry 33(5):755–762, DOI https://doi.org/10.1002/gps.4855

Murayama H, Nishi M, Matsuo E, Nofuji Y, Shimizu Y, Taniguchi Y, Fujiwara Y, Shinkai S (2013) Do bonding and bridging social capital affect self-rated health, depressive mood and cognitive decline in older japanese? a prospective cohort study. Social Science & Medicine 98:247–252, DOI https://doi.org/10.1016/j.socscimed.2013.09.026

Noguchi T, Nojima I, Inoue-Hirakawa T, Sugiura H (2019) The association between social support sources and cognitive function among community-dwelling older adults: A one-year prospective study. International journal of environmental research and public health 16(21):4228, DOI https://doi.org/10.3390/ijerph16214228

Pillemer S, Ayers E, Holtzer R (2019) Gender-stratified analyses reveal longitudinal associations between social support and cognitive decline in older men. Aging & mental health 23(10):1326–1332, DOI https://doi.org/10.1080/13607863.2018.1495178

Qiu J, Sun H, Zhong C, Ma Q, Wang C, Zhou X, Ma Y (2019) Reclassified cognitive leisure activity and risk of cognitive impairment in chinese older adults aged≥ 80 years: A 16-year prospective cohort study. Geriatrics & gerontology international 19(10):1041–1047, DOI https://doi.org/10.1111/ggi.13774

Rawtaer I, Gao Q, Zin Nyunt M, Feng L, Chong M, Shiong Lim W, Lee T, Yap P, Bee Yap K, Pin Ng T (2017) Psychosocial risk and protective factors and incident mild cognitive impairment and dementia in community dwelling elderly: Findings from the singapore longitudinal ageing study. Journal of Alzheimer's Disease 57(2):603–611, DOI https://doi.org/10.3233/JAD-160862

Sommerlad A, Sabia S, Singh-Manoux A, Lewis G, Livingston G (2019) Association of social contact with dementia and cognition: 28-year follow-up of the whitehall ii cohort study. PLoS medicine 16(8):e1002,862, DOI https://doi.org/10.1371/journal.pmed.1002862

Tang F, Zhang W, Chi I, Li M, Dong X (2020) Importance of activity engagement and neighborhood to cognitive function among older chinese americans. Research on aging 42(7-8):226–235, DOI https://doi.org/10.1177/0164027520917064

Tomioka K, Kurumatani N, Hosoi H (2018) Social participation and cognitive decline among community-dwelling older adults: a community-based longitudinal study. The Journals of Gerontology: Series B 73(5):799–806, DOI https://doi.org/10.1093/geronb/gbw059

Wang H, Jin Y, Hendrie H, Liang C, Yang L, Cheng Y, Unverzagt F, Ma F, Hall K, Murrell J, et al (2013) Late life leisure activities and risk of cognitive decline. Journals of Gerontology Series A: Biomedical Sciences and Medical Sciences 68(2):205–213, DOI https://doi.org/10.1093/gerona/gls153

Wang H, Lee C, Hunter S, Fleming J, Brayne C, Collaboration TCS (2019) Longitudinal analysis of the impact of loneliness on cognitive function over a 20-year follow-up. Aging & Mental Health 24(11):1815–1821, DOI https://doi.org/10.1080/13607863.2019.1655704

Wilson R, Boyle P, James B, Leurgans S, Buchman A, Bennett D (2015) Negative social interactions and risk of mild cognitive impairment in old age. Neuropsychology 29(4):561–570, DOI https://doi.org/10.1037/neu0000154

Zhang Q, Wu Y, Han T, Liu E (2019a) Changes in cognitive function and risk factors for cognitive impairment of the elderly in china: 2005–2014. International journal of environmental research and public health 16(16):2847, DOI https://doi.org/10.3390/ijerph16162847

Zhang W, Tang F, Chen Y, Silverstein M, Liu S, Dong X (2019b) Education, activity engagement, and cognitive function in us chinese older adults. Journal of the American Geriatrics Society 67(S3):S525–S531, DOI https://doi.org/10.1111/jgs.15560

Zhong B, Chen S, Tu X, Conwell Y (2017) Loneliness and cognitive function in older adults: Findings from the chinese longitudinal healthy longevity survey. The Journals of Gerontology: Series B 72(1):120–128

Zhou Z, Mao F, Zhang W, Towne S, Wang P, Fang Y (2019) The association between loneliness and cognitive impairment among older men and women in china: a nationwide longitudinal study. International journal of environmental research and public health 16(16):2877, DOI 10.3390/ijerph16162877
